# The microbiome of cereal plants: The current state of knowledge and the potential for future applications

**DOI:** 10.1186/s40793-023-00484-y

**Published:** 2023-03-31

**Authors:** Kristina Michl, Gabriele Berg, Tomislav Cernava

**Affiliations:** 1 Institute of Environmental Biotechnology, Graz University of Technology, Petersgasse 12, Graz, 8010 Austria; 2 Leibniz Institute for Agricultural Engineering and Bioeconomy (ATB), Max-Eyth Allee 100, 14469 Potsdam, Germany; 3 Institute for Biochemistry and Biology, University of Potsdam, 14476 Potsdam, Golm, OT Germany; 4 School of Biological Sciences, Faculty of Environmental and Life Sciences, Southampton, SO17 1BJ UK

**Keywords:** Microbiota, Microbe-host interplay, Wheat, Maize, Rice, Barley, Plant microhabitat

## Abstract

The plant microbiota fulfils various crucial functions related to host health, fitness, and productivity. Over the past years, the number of plant microbiome studies continued to steadily increase. Technological advancements not only allow us to produce constantly increasing datasets, but also to extract more information from them in order to advance our understanding of plant-microbe interactions. The growing knowledge base has an enormous potential to improve microbiome-based, sustainable agricultural practices, which are currently poorly understood and have yet to be further developed. Cereal plants are staple foods for a large proportion of the world’s population and are therefore often implemented in microbiome studies. In the present review, we conducted extensive literature research to reflect the current state of knowledge in terms of the microbiome of the four most commonly cultivated cereal plants. We found that currently the majority of available studies are targeting the wheat microbiome, which is closely followed by studies on maize and rice. There is a substantial gap, in terms of published studies, addressing the barley microbiome. Overall, the focus of most microbiome studies on cereal plants is on the below-ground microbial communities, and there is more research on bacteria than on fungi and archaea. A meta-analysis conducted in the frame of this review highlights microbiome similarities across different cereal plants. Our review also provides an outlook on how the plant microbiota could be harnessed to improve sustainability of cereal crop production.

**Supplementary Information:**

The online version contains supplementary material available at 10.1186/s40793-023-00484-y.

## Introduction

It is assumed that the plant microbiome harbours a similar potential to improve agriculture as it happened during the Green Revolution of the 60s [1]. Microbiomes not only consist of bacteria and fungi, but also include archaea, protists as well as viruses. The whole assemblage of living organisms, this excludes viruses, is termed as the “microbiota” while their genetic elements, molecular building blocks, signalling molecules, and other constituents are known as the “microbiome” as per the latest definition [2]. The emergence of high-throughput sequencing and other meta-omics technologies facilitate the assessment of microbial community compositions and functions as well as the identification of the influencing factors. Over the past years, it was shown that the plant microbiome has certain specificities that makes it clearly distinguishable from microbial assemblages that are connected to other life forms. For example, the plant rhizosphere (a term that describes the surroundings of roots that are influenced by plant exudates) provides the main environment for microbial colonization [3]. The rhizosphere is known to be highly dynamic, enriched by microbes from local soil, and subject to specific shifts associated with various plant growth stages [4]. Moreover, its colonization is highly influenced and controlled by the composition of host-specific exudates which modify the physio-chemical properties of the soil and thus the microbiome [5]. Although the role of the rhizosphere in plant health, fitness, resilience, and productivity has been known for decades, it has increasingly been recognized in recent years that the comparatively less abundant microorganisms colonizing aboveground plant tissues also have certain implications for host resilience and pathogen defence [6, 7]. Especially endophytes, which can occur inside all plant tissues, are known to be involved in a multitude of interactions with their hosts [8, 9]. There are a number of mechanisms that were identified by which microorganisms can influence plant health, stress tolerance, and productivity [10]. Bacteria can metabolize nutrients to make them available to plants (e.g. nitrogen-fixation, phosphate solubilization and siderophore production to facilitate iron uptake), induce tolerance to abiotic and biotic stress (e.g. ACC deaminase activity) or produce phytohormones (e.g. auxins) with influence on plant development [11, 12].

In the 2020 crop year, 3 trillion tons cereals were produced worldwide, whereas it is estimated that wheat, maize, and rice cultivation account for almost 90% of it [13]. In 2011, cereals accounted directly for more than 50% of the worldwide daily caloric intake, while in addition a large proportion of the grain production has an indirect influence on human nutrition through its use as livestock feed [14]. The growing world population, which is projected to be around 9.7 billion by 2050 [15], has to face the challenge of increased agricultural productivity and yield, without wasting more land and under increasingly difficult and changing climatic conditions [16]. One way to achieve these goals could be to harness intrinsic functions of the plant microbiome and its potential to promote plant growth and positively influence crop tolerance to abiotic and biotic stressors [17]. However, the integration of beneficial microbes on a large scale in modern agriculture requires a deep understanding of the underlying plant-microbe-environment interactions [18, 19].

In this review, we focus on the microbiome of the four most commonly cultivated cereal plants: wheat, maize, rice and barley [13]. Many other cereals such as sorghum, rye, triticale, oats and millets as well as the so-called pseudocereals including amaranth, quinoa, buckwheat and various others are less commonly grown; they were not considered in this review. It is also noteworthy to mention that most of these plant species have several varieties and a scarcely assessable number of cultivars [20, 21]. In order to summarize current knowledge related to the microbiome of cereal plants, we performed an extensive literature search with the main databases PubMed and Google Scholar in the period of 02 February 2021 until 13 July 2021. The keywords “wheat”, “*Triticum*”, “maize”, “corn”, “*Zea*”, “rice”, “*Oryza*”, “barley”, “*Hordeum*”, and “cereals” were combined with either “microbiome” or “microbiota”. Several additional articles were found by searching for reviews and references that were cited within the articles that were found during the initial search. Studies not based on high-throughput sequencing and reviews were excluded to achieve greater consistency. A total of 302 articles were selected, all of which were published between 2013 and 2021. A table (Supplementary Table 1) with the microorganisms (bacteria, fungi and/or archaea) discussed in the articles, the plant compartment (soil, rhizosphere, roots, aboveground plant compartments, and specifically seeds) as well as the general topic of all included research articles was compiled during the preparation of the review. One point was given for every subject matter of the table that was addressed in the articles; for example, if a research article contained data on the microbiome of both wheat and maize, each subject matter received one point. Many studies contained more than one study matter, thus dividing through the total amount of publications would result in percentage numbers higher than 100%. We therefore decided to divide through the total number of entries in the compiled table. The same strategy was also applied for plant microhabitats and microorganism within the domains bacteria, fungi, and archaea. However, for simplicity, we always refer to “articles” and not the number of entries throughout the review. A general overview is provided in Table 1, while detailed information can be found in Supplementary Table 1. Overall, this review is meant to provide a comprehensive overview of the current knowledge base and to highlight key findings of the last years that were inferred from microbiome studies of cereal plants.

**Table 1:** Overview of the general topics in studies assessing the microbiome of cereals. The numbers in brackets represent the absolute numbers of entries for the specific topics, while the percentages were calculated by dividing absolute numbers by the total number of entries

Topic	Wheat	Maize	Rice	Barley
Total number of papers	103	101	91	23
Total number of entries	164	155	143	40
**Comparative assessments**	32.3% (53)	31.0% (48)	36.4% (52)	40.0% (16)
**Agronomic management**	15.2% (25)	14.2% (22)	11.2% (16)	10.0% (4)
**Fertilizers**	9.1% (15)	16.8% (26)	4.9% (7)	7.5% (3)
**Environmental impacts**	13.4% (22)	7.7% (12)	11.9% (17)	12.5% (5)
**Soil contaminations**	1.8% (3)	1.3% (2)	9.1% (13)	5.0% (2)
**Plant metabolites**	1.2% (2)	4.5% (7)	2.1% (3)	0
**Abiotic stress**	4.3% (7)	1.9% (3)	2.8% (4)	7.5% (3)
**Transgenic plants**	0	2.6% (4)	2.1% (3)	0
**Evolution, Transmission, Breeding**	7.9% (13)	5.2% (8)	7.0% (10)	10.0% (4)
**Pesticides**	4.9% (8)	2.6% (4)	3.5% (5)	2.5% (1)
**Pathogens**	4.9% (8)	4.5% (7)	4.2% (6)	2.5% (1)
**Biocontrol and Biostimuli**	4.9% (8)	7.7% (12)	4.9% (7)	2.5% (1)

### Recent findings related to the wheat microbiome

Wheat (plants from the genus *Triticum* L.) globally accounts for the largest cultivation area of all cereal plants. In the last 20 years, the area under cultivation for wheat has remained constant, while the yield steadily increased. Wheat is mainly cultivated in the European Union, China, and India, accounting for over 50% of the global wheat production [13].

**Fig. 1:**
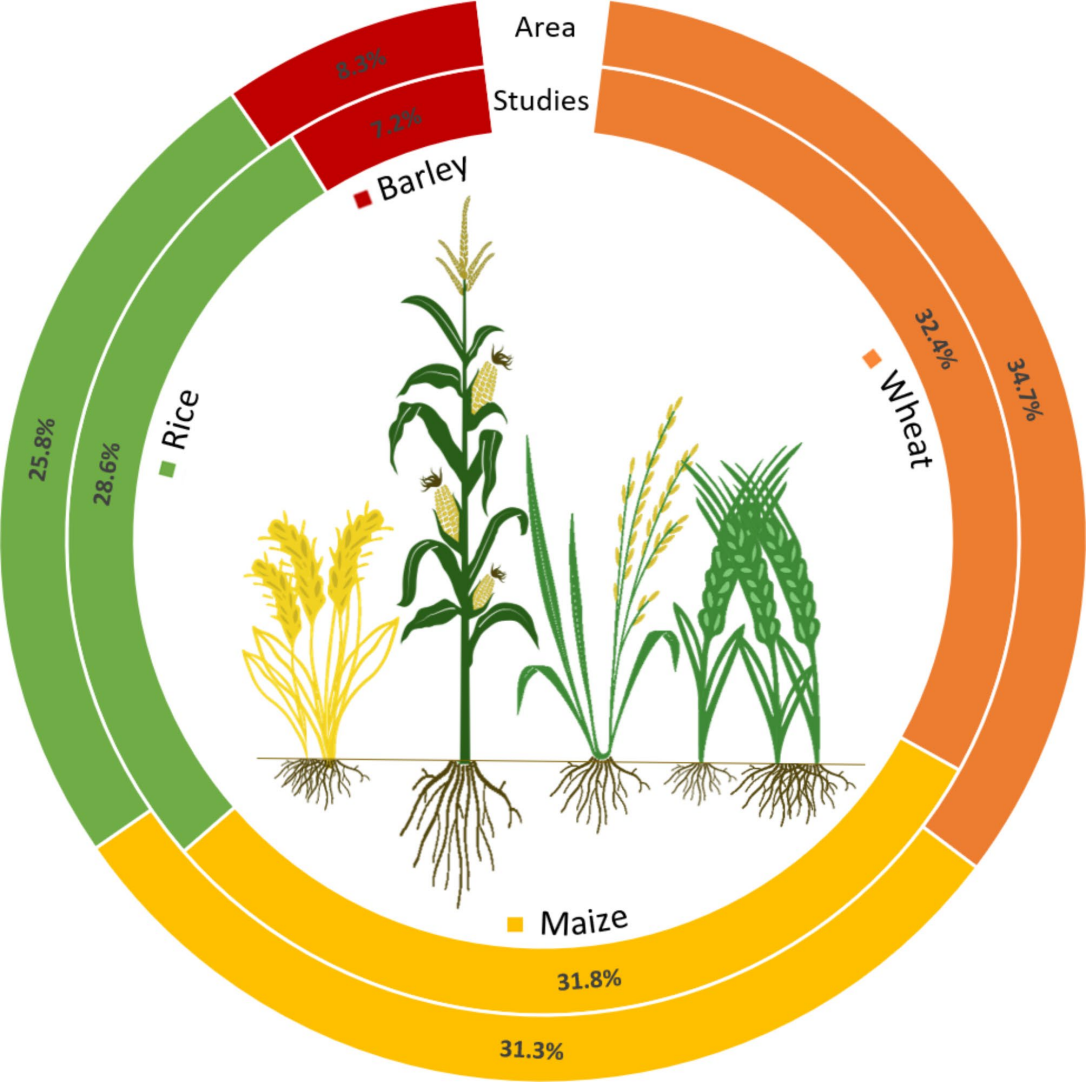
Cultivation areas of globally prevailing cereal crop plants and the number of corresponding microbiome studies. The cultivation areas are shown in the outer ring as percentages of the total area (622 million ha) used to cultivate the four crops [9]. The inner ring indicates the percentage of microbiome studies obtained by extensive literature search within publicly accessible databases

**Fig. 2:**
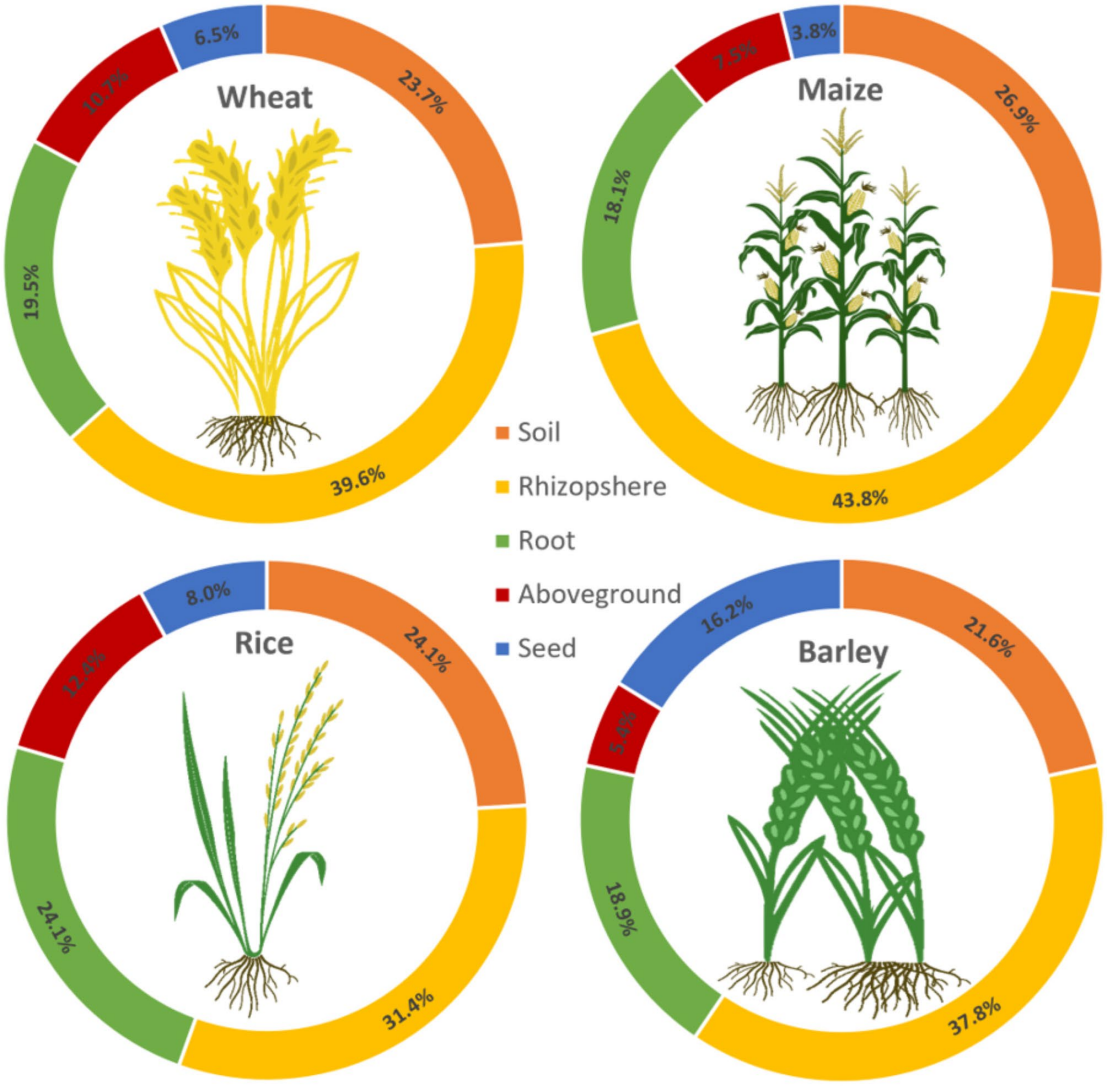
Proportions of studies addressing the microbiome of specific plant compartments. The belowground parts are most commonly addressed in the currently available studies for all major cereal plants. This is followed by the aboveground parts and studies that are specifically focused on seeds; except for barley for which more studies focus on the seeds than on other aboveground compartments

**Fig. 3:**
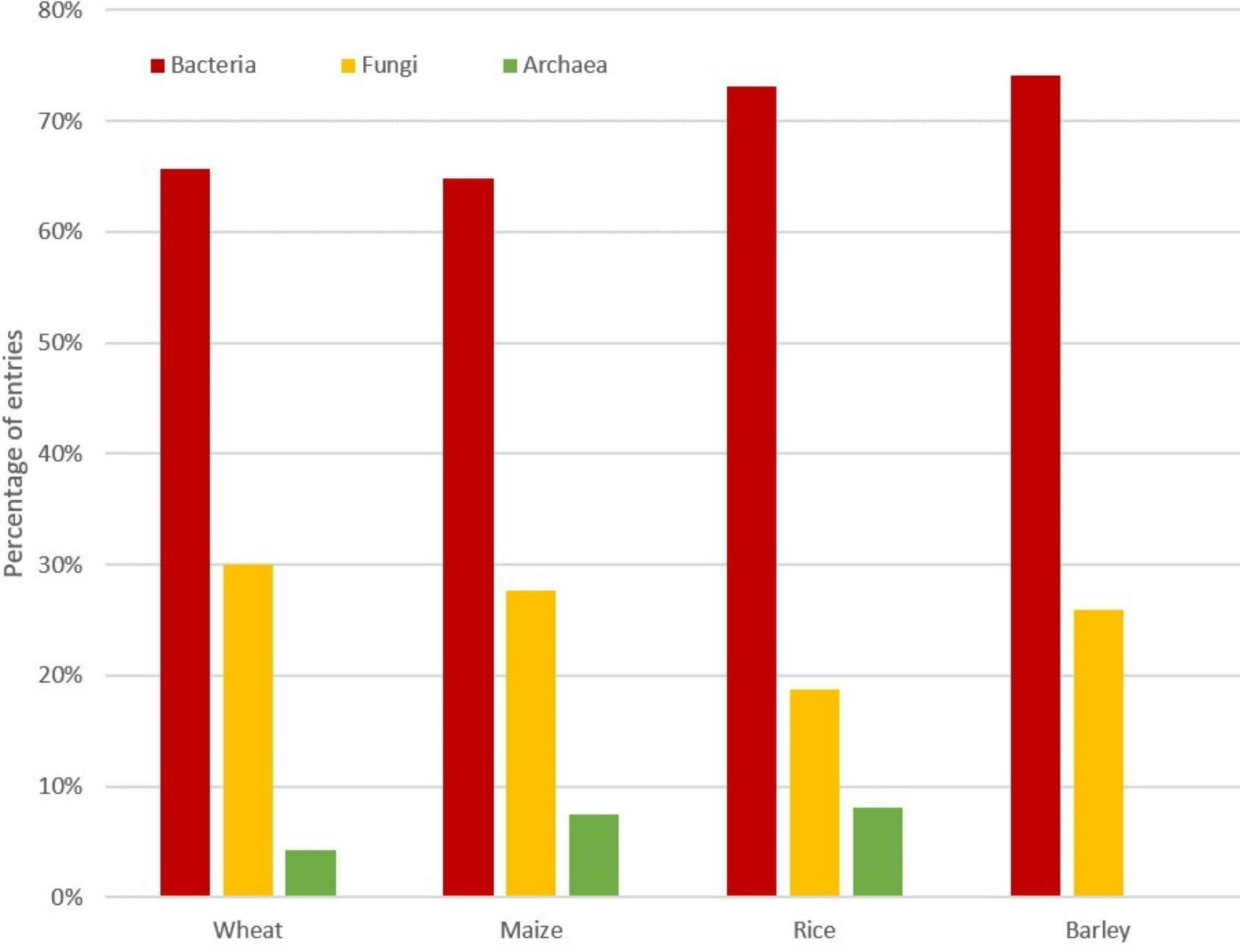
Proportions of microbiome studies targeting certain organism groups in microbiomes. Bacterial communities are most commonly addressed in the currently available studies for all major cereal plants. This is followed by fungi and archaea, which account for a substantially lower number of studies

From the 302 assessed articles, the largest fraction (32.4%; Fig. 1) included data related to the wheat microbiome. The majority of these articles focused on the below-ground microbiome, including soil, rhizosphere, and roots, accounting for 23.7%, 39.6% and 19.5%, respectively (Fig. 2). The aboveground microbiome was addressed in 17.2% of the studies assessed, with 10.7% addressing shoots, leaves and/or stems; 6.5% specifically addressed the plant’s seeds. Bacteria were more often in the focus of the research than fungi and archaea, with 65.7%, 30.1% and 4.2%, respectively (Fig. 3).

The main research objectives related to the differences in the microbiome compared to other plant species, genotypes, plant compartments, and developmental stages (Table 1). It was shown that all these factors have a certain influence on the microbiome, although to varying degrees [22–26]. The rhizosphere, for example, was commonly shown to differ from the bulk soil. Although the bacterial diversity was lower than in soil, the rhizosphere harboured microorganisms that were interconnected in less complex, but more stable co-occurrence networks and characterized by a more stable diazotrophic community structure [27, 28]. These observations reflect general characteristics of the rhizosphere that are commonly observed within the plant kingdom [29, 30]. Chen et al.[26] showed that the plant developmental stage had a stronger impact on the bacterial community than on the fungal community composition of wheat plants. Moreover, they observed that the abundance of plant growth-promoting rhizobacteria (PGPR) under high inorganic nitrogen (N) fertilization correlates with the root-released organic carbon levels, which were subjected to certain dynamics during developmental stages, as they are higher in the jointing and ripening stages compared to the tillering stage. They proposed that recruitment of beneficial microbes to cope with high N inputs is a controlled mechanism regulated by the host plant through the secretion of organic acids. Several more studies addressed the influence of different fertilizers and their management on the wheat microbiome. The most common outcome was that fertilizers have an impact on the microbiome, which depends on the fertilization method [31–33]. Wang et al.[31] found that soil microorganisms show different responses to inorganic and organic fertilization, which results in clearly differentiable communities. Further, Kavamura et al.[32] showed that inorganic fertilization has a negative impact on rhizosphere bacteria with a less diverse, rich and stable community compared to organic treatments. The impact of agricultural practices, mainly crop rotation, tillage, and management type, on the microbiome is another major topic that was addressed in wheat microbiome studies. In contrast to studies that focused on fertilization practices, there was a higher variability in terms of results related to the extent of the impact of agricultural practices on the belowground microbiome. Hartmann et al.[34] showed that tillage and management type do not affect the richness, but the composition and structure of microbial communities in soil and plant roots. It was furthermore shown that agricultural intensification negatively influences the abundance of keystone fungal taxa and reduces the root fungal network connectivity [35]. On the other hand, Lupwayi et al.[36] found that neither tillage nor crop rotation had an effect on the alpha diversity and the relative abundance of the rhizobacterial community. Studies that focused on the plant genotype indicated that the microbiome of domesticated wheat plants is different in comparison to their ancestors and wild varieties [37, 38]. The authors provided evidence that they follow distinct community assembly strategies and that wild plants harbour more structured and more defined communities, yet they are less diverse compared to domesticated cultivars [39, 40]. In terms of agrochemical treatments, a high degree of specificity in terms of the applied substances was observed. Schlatter et al.[41, 42] showed that glyphosate had only limited effects on bacterial and fungal communities, while Qu et al.[43] observed that the herbicide S-metolachlor substantially influenced the microbial richness in the rhizosphere. Interestingly, they found that the proportion of distinct, beneficial bacteria can increase upon treatments, proposing a plant-regulated mechanism to cope with herbicide stress. In addition to the above-mentioned studies, there are also several studies that focused on the influence of pathogens, biocontrol agents, as well as different biostimuli on the microbiome. Seybold et al.[44] identified a correlation between changes in the wheat leaf microbiome and the suppression of immune-related metabolites by the pathogen *Zymoseptoria tritici*. Chen et al.[45] showed that the wheat-associated bacterium *Pseudomonas piscium* secrets a compound which suppresses growth and virulence of the fungal pathogen *Fusarium graminearum* by targeting a histone acetyltransferase and thereby dysregulating histone acetylation. Overall, several trends in wheat microbiome studies have become evident in recent years. Much emphasis was placed on the general assessment of the microbiome and to identify factors influencing microbial community compositions.

### Microbiome-related discoveries in maize

Maize (plants from the genus *Zea* L.) globally accounts for the second largest cultivation area, with 0.9 hectares (ha) maize crop area per 1 ha wheat crop area. The crop area has increased constantly by 1.4 times over the last 20 years, while the harvest volume has doubled [13]. The by far leading corn-producing country is the US. It produced 31% of the world’s harvest in the harvest year 2019 and up to 91% of the grown plants were genetically engineered [13, 46]. About a third of the corn production in the US is used for feeding livestock, another third serves as main source for production of fuel ethanol. The rest is exported and used for human food and beverages as well as industrial applications, like packing material or insulin [47]. In comparison to wheat, rice and barley, maize is a C4-plant; these plants avoid photorespiration and have a higher water use efficiency. These host characteristics may be involved in shaping their microbiota.

The second largest proportion of the studied articles (31.8%; Fig. 1) included data on the maize microbiome; this is only 1.85% less than for wheat. The majority, 88.8%, of the research articles focused on the belowground microbiome, including soil, rhizosphere and roots, accounting for 26.9%, 43.8% and 18.1%, respectively (Fig. 2). The above-ground microbiome was addressed in 11.3% of the research articles, while 7.5% and 3.8% focused on the shoots and kernels, respectively (Fig. 2). Bacteria were again the focus of research (64.9%; Fig. 3), at the expense of research on fungi, which were covered in only 27.7% of the articles.

The main research objectives also related to differences in the microbiome compared to other plant species, genotypes, compartments or developmental stages. In accordance to wheat, it was shown that all these factors can have impacts on the microbiome[22, 48, 49] (Table 1). However, there is more research on the influence of fertilizers and their application strategies on the maize microbiome, compared to the other cereals (Table 1). Xiong et al.[22] found that the cultivation site and fertilization practice have a lower impact on microbiome assembly than the plant compartment and host species. Moreover, they found that host selection increases while bacterial diversity simultaneously decreases from soil to epiphytes to endophytes. Compared to wheat, we observed that there are more research articles that focus on the impact of plant metabolites, especially benzoxazinoids (BXs), on the microbiome. We hypothesize that this is due to the fact that the BX biosynthesis pathway was decoded in maize, and thus provides a tangible link to the microbiome that can be integrated by studies. BXs are only produced by distinct plant species, including major agricultural crop plants like maize, wheat, and barley; however, BXs are not present in rice [50]. Kudjordie et al.[49] showed that the genotype effect on microbial communities is stronger in roots than in the rhizosphere and that plant pathogens negatively correlate with the secretion of BXs. These metabolites also appeared to have a greater impact on the fungal richness than on the bacterial. Moreover, Hu et al.[51] showed that BXs influence herbivore defence of the next generation of plants by changing the root and soil microbiome. There are two studies that highlight *Trichoderma* (*T. harzianum* and *T. asperellum*) as potential biocontrol agent (BCA) to control stalk rot caused by *Fusarium graminearum*. Both studies reveal differences in the microbiome community and disease reduction upon application of the BCA [52, 53]. In several other studies, it was shown that agricultural practices highly influence the maize microbiome [54, 55]. Schmidt et al.[56] found that recruitment of microorganisms differs between agricultural management types and that bacteria and fungi respond differently to the management type. They showed that microbial communities in the soil and rhizosphere respond differently to management strategies and propose that roots should be considered as a crucial factor influencing management outcomes. Ares et al.[55] compared two different maize genotypes (SinPre and Pigarro) under conventional and organic management and found that higher microbial diversity was associated with organic farming, as was the presence of AMF. Furthermore, it was shown that crop rotation had a bigger influence on the fungal soil community, than on the bacterial [54, 57]. Overall, recent studies found that the maize and wheat microbiome had many characteristics in common.

### Summary of ongoing rice microbiome research

Rice (plants from the genus *Oryza* L.) globally accounts for the third largest cultivation area, with 0.75 ha rice crop per 1 ha wheat crop area. China and India are the main rice producers, accounting for over 50% of the global rice production in the harvest year 2019 [13].

From the 302 studied research articles, the third largest fraction (28.6%; Fig. 1) included data related to the rice microbiome. The majority, with 79.6%, of the assessed studies, focused on the belowground microbiome, with 24.1%, 31.4%, and 24.1% on the soil, rhizosphere, and roots, respectively. The aboveground microbiome was the focus of research in 22.4% of the articles, with 8% addressing seeds and 12.4% shoots (Fig. 2). When the collected literature was assessed, we noticed that the rhizosphere microbiome was less frequently (31.4%) explored compared to wheat and maize (39.6% and 43.8%, respectively) and that more emphasis was put on the root microbiome (24.1%). With 73.2% of the articles, bacteria were again studied more frequently than fungi with 18.7% and archaea with 8.1% (Fig. 3).

The main research objective addressed was targeting differences in the microbiome, as also observed for wheat and maize. Other frequent objectives included comparisons to other plant species, genotypes, developmental stages, or plant compartments (Table 1). As with wheat, the influence of agricultural practices on the microbiome was the second most addressed topic. A major focus was on the effects of different irrigation methods on the below-ground microbiome [58–62]. Compared to research on wheat and maize, there is less research on the impact of fertilizer use on the rice microbiome and more on the influence of soil pollution with pesticides and heavy metals, like methylmercury, arsenic, or antimony. Liu et al. [63] showed that the soil microbial community was able to quickly adapt to exposure to the three commonly used pesticides butachlor, clothianidin and tricyclazole. They found only minor changes in community composition and diversity; the differences even decreased over the growth period. Moreover, Chen et al. [64] and Qian et al. [65] showed that root exudation in rice plants increased upon exposure to the herbicide diclofop-methyl, leading to increased bacterial biomass, richness, and diversity. They hypothesized that it was possibly due to a protection mechanism of the rice plant as a response to the herbicide treatment. Moreover, it was shown several times that the rhizosphere and soil microbiome respond to heavy metal contamination. Recently, Das et al. [66] proposed fertilization with silicate as a possible mechanism to improve bacterial stress tolerance in arsenic-polluted soils. Microorganisms can substantially influence the solubility of pollutants and bio-transform (e.g. methylation, oxidation and reduction) them. Interestingly, this process has been shown to be strongly influenced by water management in rice cultivation, probably due to the different redox potentials of aerobic and anaerobic soils [59, 67, 68]. Water management in general has been shown to significantly affect the rice microbiome, and distinct bacterial communities were found in flooded and non-flooded rice fields [60]. A distinct shift in microbial communities towards more consistent compositions was also observed in extensive rice monocultures. Interestingly, it was demonstrated that this is only partly due to agricultural management, but that there is also a strong influence of the plants themselves, leading to an enrichment of distinct taxa in rice fields, e.g. methanogenic archaea [69]. In terms of rice seeds, most of the studies focused on the description of microbial communities in different host genotypes. Recently, however, a transgenerational mechanism via the seed microbiota to confer resistance against *Burkholderia plantarii* was discovered. The seed-endophytic bacterium *Sphingomonas melonis* was identified as a key player in the tripartite interaction. It confers disease resistance to a broad range of rice genotypes by producing anthranilic acid which impairs the virulence factor biosynthesis of *B. plantarii* [8].

### Recent discoveries in the barley microbiome

Barley (plants form the genus *Hordeum* L.) globally accounts for the fourth largest cultivation area, with 0.23 ha barley crop per 1 ha wheat crop area. The European Union is the leading barley-growing region, accounting for over 40% of the global barley production [13]. From the 302 assessed articles, the by far smallest fraction (7.2%; Fig. 1) included data related to the barley microbiome. As with the other cereal plants, the majority of the articles focused on the below-ground microbiome (78.3%; Fig. 2). The aboveground microbiome was addressed in 21.6% of the studies, with a specific focus on the plant’s seed microbiome (16.2%, Fig. 2). Most of the studies focused on bacteria (74.1%, Fig. 3), and only to a lesser extent on fungi (25.9%; Fig. 3), while no studies were found that addressed archaea.

The vast majority of the assessed publications presented data related to differences in the microbiome compared to other plant species, genotypes, plant compartments and developmental stages (Table 1). Bulgarelli et al. [70] showed that the barley genotype has a rather small but significant impact on the root microbiome and that the community composition is influenced by the combined effect of microbe-microbe and plant-microbe interactions. Yang et al. [71] found that plants grown in autoclaved soil, which simulated a disturbed microbiome, were severely affected by additional drought stress. The reduction in biomass was connected with major changes in the microbial community composition, which indicated a shift in plant colonization from soil-derived bacteria to seed-originated endophytes. Moreover, plants grown in field soil showed a significantly higher potential to be resistant against *Blumeria graminis* and lower infection rates compared to plants grown in potting soil. The authors hypothesized that this was due to the fact that field soil harbours higher microbial diversity which allows enrichment of beneficial bacteria [72]. Rahman et al. [73] found that the barley seed microbiome harbours several beneficial strains and inoculation with them promoted, not only plant growth, but also resistance against *Blumeria graminis.* The barley seed microbiome is, compared to the other cereals, more in the focus of research. Abdullaeva et al. showed that cultivated barley (as well as wheat) has a more diverse seed microbiome, including bacterial taxa linked to the human microbiome, than its wild ancestor *Hordeum spontaneum*, yet it is less connected. Furthermore, they could detect indications for co-evolution between the plant and their microbiome during the domestication process [40].

### Common features of cereal crop microbiomes

The proportion of microbiome studies on the four cereal crops corresponds relatively closely to the proportion of their cultivated area, with only rice being slightly overrepresented and barley being slightly underrepresented (Fig. 1). Although the microbiome of each of the cereal crops discussed here has its own characteristics, certain commonalities among the hosts have been identified. It is particularly noteworthy that in all four crops, the belowground microbiome and bacterial communities have been the focus of research in recent years. All four cereal plants had in common that they harboured *Proteobacteria*, *Actinobacteria*, *Firmicutes*, *Bacteriodetes*, *Acidobacteria*, and *Chloroflexi* in their microbiome as prevalent bacterial phyla. Reviews by Kavamura et al. [74] and Mehta et al. [75] already presented the most commonly detected bacterial genera in the wheat and maize microbiome, respectively. *Pantoea*, *Pseudomonas*, *Rhizobium*, *Sphingomonas*, and *Stenotrophomonas* constitute some of the bacterial genera that are commonly found in both wheat and maize. When the literature related to rice and barley was assessed, we found that these bacterial genera also commonly occurred in their microbiome.

Many of the conducted studies led to the identification of beneficial bacteria and biostimulants which refers to microorganisms with plant growth-promoting, disease-supressing, and/or other fitness-enhancing traits [76]. Interestingly, the majority of the identified strains was assigned to the bacterial phyla *Proteobacteria* and *Firmicutes* and the fungal phyla *Ascomycota*, more specifically the genus *Trichoderma*. Members of the genera *Pseudomonas* and *Bacillus*/*Paenibacillus* were often identified as plant-beneficial bacteria. For example, *Pseudomonas stutzeri* inoculation increased plant development and had a positive impact on bacterial community composition, particularly among diazotrophs and ammonia-oxidizers [77]. Similar results were obtained by Li et al. [78], showing that inoculation with *Paenibacillus triticisoli* led to increased dry maize biomass and a shift in the microbial community, especially in a low nitrogen environment. They both demonstrated that biostimulants not only promote plant growth by the strain’s specific characteristics, like nitrogen fixation, but can also modulate the microbiome.

Recent advances in microbiome-related techniques allow us to analyse microbial communities in detail, estimate their complexity, and study their interplay with host plants; however, most of the studies conducted to date have dealt exclusively with bacterial communities [79, 80]. In the future, increasing the proportion of research on fungi, archaea, and protists in cereal crops will be crucial to fully understand the relationship between plants, microbes, and the environment. So far, studies targeting fungal communities have shown that cereals are dominated by *Ascomycota* and to a lesser extent by *Basidiomycota* [45, 71, 73–75, 81–91]. Archaea were found to be the focus of the lowest proportion of the reviewed studies. Furthermore, archaea are only considered in metagenomic /-transcriptomic studies (38%) or included in 16S rRNA gene analyses conducted with bacteria-specific primers (38%). Only a few studies are based on archaea-specific primers (23%), which may lead to an incomplete picture of the archaeal community composition. Rice cultivation is the cause of 1.3% of the global greenhouse gas emission, because its cultivation in flooded paddies results in anaerobic soil conditions, which provide optimal growing conditions for methanogenic archaea [92]. For this reason, a major fraction of research on archaea is done in rice paddies and to a lesser extend in maize and wheat. In wheat the most commonly detected archaeal phylum was *Nitrososphaerota* and to a lesser extend *Euryarchaeota* [27, 93, 94]. However, in rice, the phyla *Euryarchaeota* and *Crenarchaeota*, including the methanogenic archaea, were detected most commonly [95, 96]. There is a substantial variation in archaeal community compositions between the studies, which may have different reasons [62, 85, 95, 96]. For one, only a low number of studies is available and in addition they focus on different compartments, treatments, and other factors. Furthermore, the use of different primers to assess the community may lead to substantial variations.

**Fig. 4:**
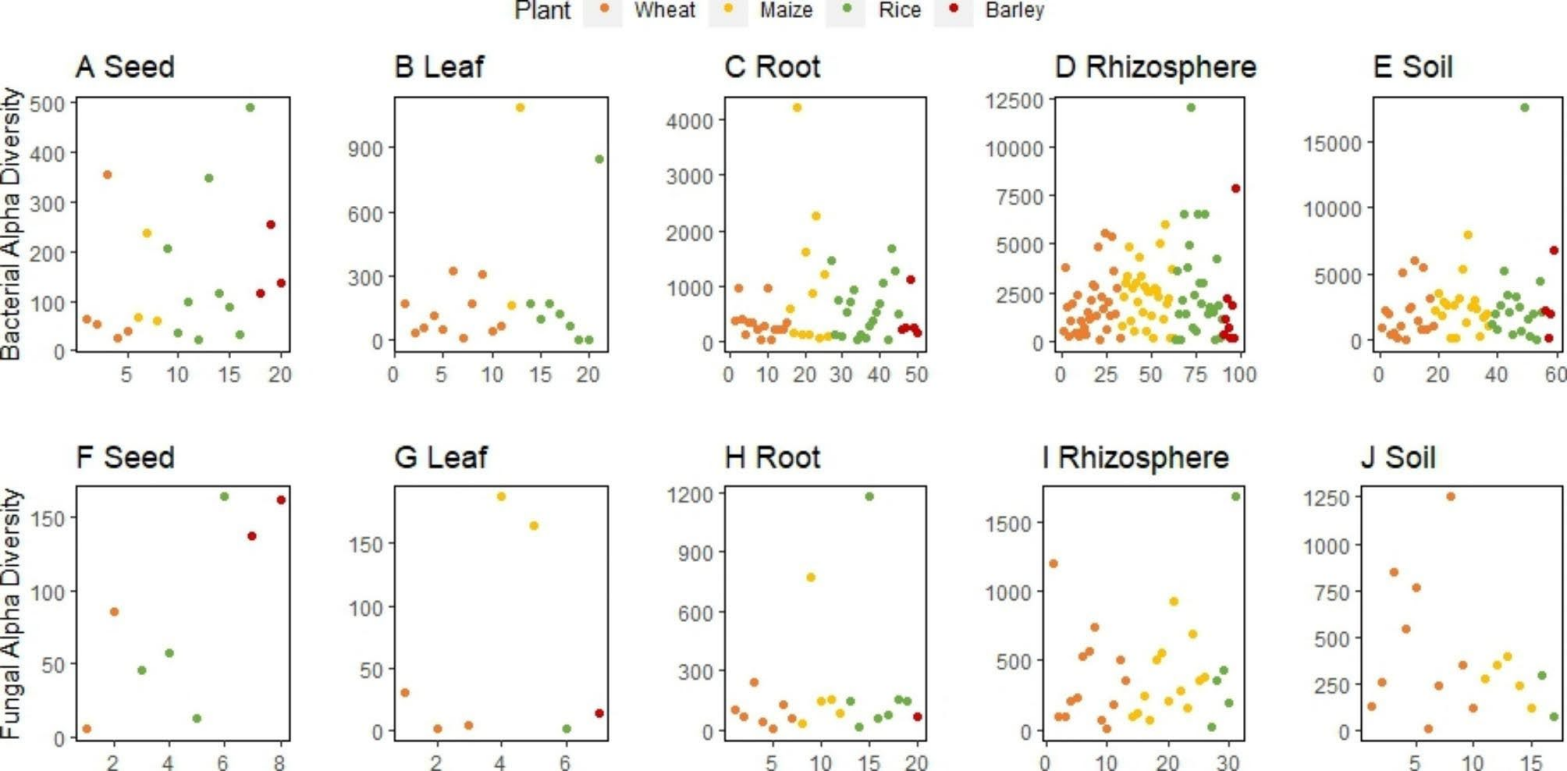
Assessment of bacterial (A-E) and fungal (F-J) diversity in the different compartments of cereals. The diversity numbers were extracted as Chao1 index or ASV richness from the manuscript text or plots. The diversity in each compartment is not significantly different between the cereals

A meta-analysis conducted in the frame of this review indicates that there is no significant difference in microbial diversity between the four plant types in any compartment (Fig. 4). The utilized data were extracted from 160 manuscripts depicting alpha diversity and the methods are further described in the Supplementary Information. Certain tendencies were observed in the meta-analysis, yet there is substantial variability across the studies. This observed variability could be due to several reasons, e.g. different primer sets used to amplify marker genes, differences in sequencing platforms, non-standardized protocols to extract total community DNA, etc. Furthermore, there are substantial differences in relation to the sampling process, especially concerning (bulk) soil. We noticed that the definition of bulk soil is very broad. It ranges from soil collected from neighbouring fields to soil only loosely adhering to the plant. Soil samples not sampled in the field at least during cultivation, or from pots not planted were excluded in our meta-analysis for better consistency. A standardized protocol as proposed by Barillot et al. [97] could help overcoming these variations. In order to be able to clearly differentiate between bulk-, rhizosphere-, and rhizoplan soil fractions they suggest a 3-step protocol, starting with vigorously shaking the roots to remove loosely bound soil considered as bulk soil. Subsequently, the rhizosphere is sampled by shaking roots in a NaCl solution to catch the soil directly adhering to the roots. Finally, roots are washed and again shaken in a solution containing NaCl and Tween to collect the rhizoplane fraction. However, it will require host-specific adaptations because this protocol was established for small, herbaceous plants and may not be directly applied to crop plants grown under different environmental and soil conditions.

There were no observable differences in microbial diversity of rhizosphere and soil samples, but the soil compartments were substantially higher compared to endosphere samples. The endorhiza was generally more diverse than endophytes found in the aboveground parts of the host plants (Fig. 4). It is important to highlight that not all samples from the endosphere were surface-sterilized. Meta-analyses of fungal communities showed similar results. The diversity in the soil compartments was much higher than in roots, leaves and seeds (Fig. 4). Interestingly, the intensely bred cereal plants maintained the evolutionary old trait to form arbuscular mycorrhizal symbioses, which offers a great potential for enhancing yield without using synthetic fertilizers [98]. However, due to the fact that less data is available for fungi than for bacteria, the analysis might be biased to a certain degree.

**Fig. 5:**
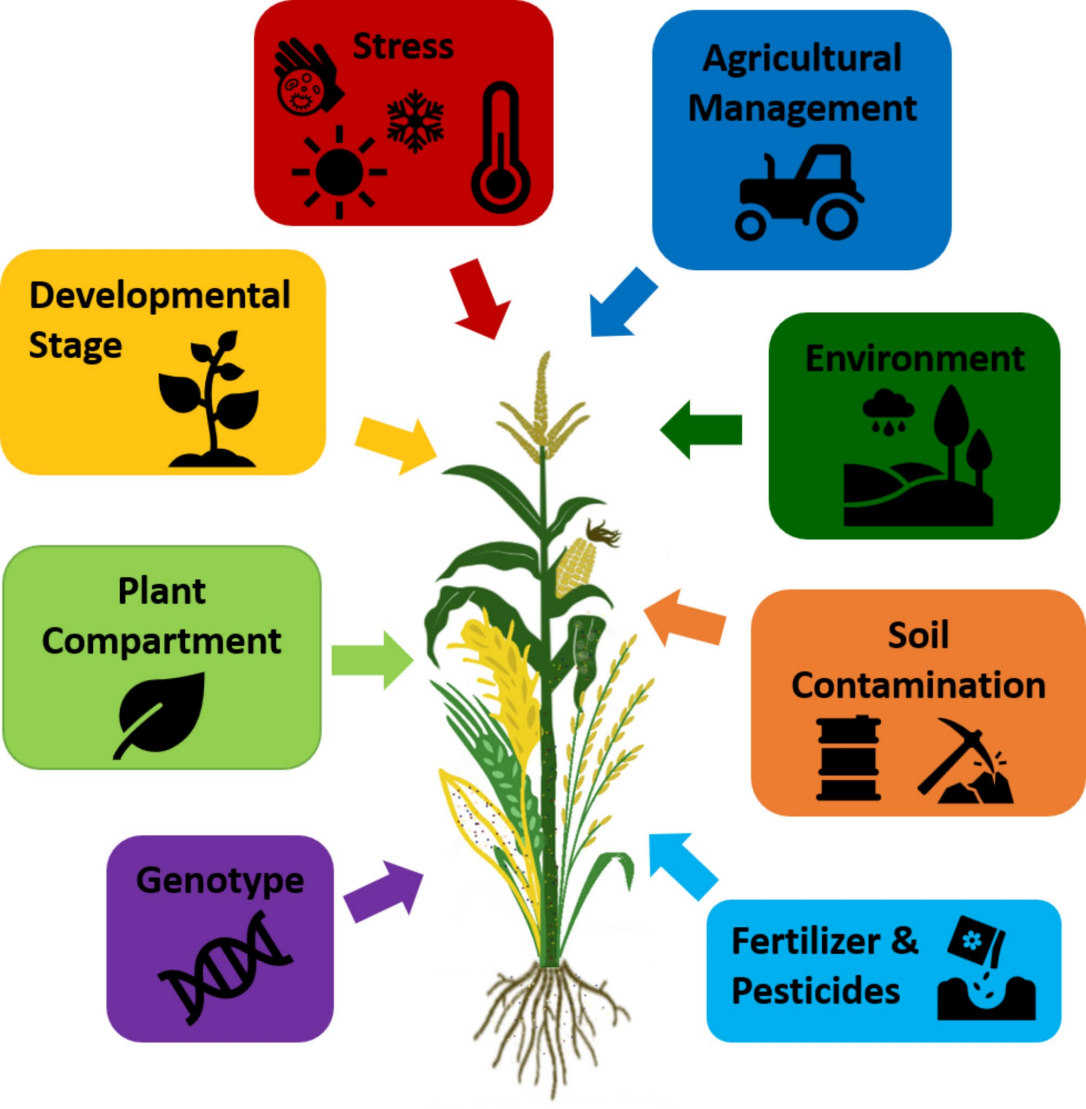
The plant microbiome is influenced by various factors. These influencing factors can be intrinsic (e.g. genotype, compartment and developmental stage) or external factors, including agricultural management or abiotic and biotic stress

The overall microbiome-related objectives addressed in the assessed studies were similar for all four plants. The most commonly addressed topic was the general and comparative assessment of their microbiome, followed by agronomic practices (e.g. tillage, organic vs. conventional management, crop rotation). Differences were observed in terms of the number of studies focusing on fertilization management, which was more often in the focus for maize studies compared to the other cereals. Moreover, effects of soil contaminations were mostly addressed in rice microbiome studies. Overall, it is clear that there are many factors that influence the composition of the cereal plant microbiome; the major studied factors are depicted in Fig. 5. Interestingly, the assessed studies had only a minor focus (4.1% on average) on the impact of abiotic stress (e.g. water stress) on the microbiome and how the microbiota could be harnessed to protect host plants under these conditions. In addition, the influence of plant pathogens on the microbiome was comparatively less discussed (4% on average) in the reviewed literature. Several studies addressed the overall change in the microbiome composition when plants are confronted with pathogens [44, 45, 72, 99]. Bacterial communities, including such that promote plant growth, showed a tendency to be more diverse when a pathogen was present [99–102]. It is generally known that moderate disruptions can cause diversity increases in various ecosystems, however, lasting disturbances by plant pathogens (and abiotic stress) are mostly linked to adverse effects [103].

It should be highlighted that many of the described commonalities between the four addressed crop plants are also present in various other plant species, therefore they are not only distinct to cereal crops. Moreover, it can be expected that a growing number of available datasets combined with big data analyses will allow us to better understand the underlying factors of similarities and differences in the future.

### The potential of the cereal crop microbiome for future applications

The current state of knowledge indicates that functions provided by distinct microbial communities associated with plants can be harnessed to reduce the use of agrochemicals and fertilizers [104]. This is especially important for cereal crops, because they not only globally account for the largest cultivation areas, but also provide staple food for over half of the world’s population. Agriculture is an important factor in achieving climate goals of the European Union and beyond, as it has been identified as one of the main causes of climate change. The recently presented Farm to Fork Strategy as part of the European Green Deal has the ambitious goals to halve the use of pesticides, reduce overfertilization and triple the area of sustainable agriculture for a fair and environmentally friendly food system as well as to counteract the immense biodiversity loss and depletion of natural resources [105]. It is estimated that pre-harvest grain loss due to biotic factors, like pests, pathogens and weeds, or abiotic stress, can account for up to 35% of the total harvest. Another 20% is globally lost during storage and affected by growth of mycotoxigenic fungi which can also lead to a decline in grain quality [106]. Solanki et al. [107, 108] showed that traditional methods to eliminate insects before storage, like seed fumigation, have a non-target effect on the microbiome. These applications decrease bacterial diversity over time. Furthermore, they isolated several bacterial and fungal strains from the wheat seed microbiome with the potential to reduce fungal and mycotoxin contamination. The plant microbiome will likely play an important role in providing solutions for sustainable agricultural practices; previous research has demonstrated that various members of the indigenous microbiome of cereal plants can be used to increase yields while simultaneously reducing emissions as well as the use of agrochemicals and fertilizers. During the last years, several microbial strains were identified in cereals that might be suitable to substitute pesticides to combat major plant diseases. It was shown that *Pseudomonas piscium* and *Pantoea agglomerans* can be used against Fusarium head blight in wheat, *Sphingomonas melonis* for rice seedling blight, and *Enterobacter cloacae* to fight stalk and ear rot in maize [8, 45, 72, 109, 110]. Furthermore, there are instances where PGPR strains have been applied as BCA, offering a dual advantage of both reducing disease incidence and stimulating plant growth [52, 111–113]. In terms of fertilizer reduction, one recent study provided an important link between rice genetics and the enrichment of nitrogen cycle related bacteria in the plant’s rhizosphere. Zhang et al. provided evidence that the presence of natural variation of the nitrogen transporter NRT1.1B in the *indica* rice varieties is associated with the recruitment of a root microbiome with functions related to the nitrogen cycle, and especially to the ammonification process [114]. In the future, these findings could be used to selectively breed plants that can establish and maintain a functional microbiota that improves the plant’s nitrogen-use efficiency in order to reduce fertilizer inputs. Moreover, several bacterial strains with PGP traits were identified in cereal crops that may improve plant growth, especially under abiotic stress. For example, *Bosea* sp. and *Pseudoduganella* sp., both isolated from maize, promote growth of juvenile plants under cold temperatures while *Curtobacterium flaccumfaciens*, isolated from drought tolerant wheat, was shown to significantly increase plant growth under adverse conditions [115, 116]. The application of microbial inoculants is highly complex and their successful introduction depends on several factors, including the specific strain, application mode and time as well as the host microbiome itself [117]. Therefore, further research will be required to identify inoculants that are compatible with the host plant as well as its microbiome under distinct, and often adverse, conditions. Although research on wheat, maize, and rice is already tremendous compared to other plants, especially concerning the general assessment of the microbiome, there is still a lack of information on the functional aspects of the microbiome and how it can be specifically manipulated and engineered to improve plant productivity and health. Furthermore, as previously highlighted, cereal microbiomes share many common characteristics, suggesting that many findings obtained with a particular plant species may be transferable to others.

## Propositions for further consideration

1. A large number of studies addressing microbiomes of cereal crops were conducted in recent years; however, they are mainly based on the analysis of a specific plant compartment. More research based on holistic approaches covering the whole plant will be essential to improve our understanding of microbiome assembly and dynamics.
2. The majority of available studies focuses on belowground plant compartments. Aboveground compartments, and especially seeds, are currently understudied. Recent research has indicated that seeds might harbour highly effective biostimulants, plant growth promoters, and biocontrol strains [118].
3. Climate change with more frequently occurring weather extremes like drought, heavy rainfalls, and in more general, conditions allowing pathogens to proliferate, highlights the importance of research on the response of the plant microbiome to abiotic and biotic stress. This will allow to identify key players to increase plant resistance to different stress factors. In addition, more studies on the effects of combined stresses, which are characteristic for the Anthropocene, on the plant microbiome and plant performance are necessary.
4. Many agrochemicals were shown to have off-target effects on naturally occurring microbial populations; this also includes antimicrobial resistance formation. Most of the current studies focus on resistance formation in target organisms, but ignore the effect on the overall resistome. Future studies should subject agrochemicals to deepening analyses which includes the evaluation of representative plant resistomes.
5. Most of the ongoing microbiome research is focused on bacteria, followed by fungi. The other constituents of the microbiota, including archaea, protists, and algae, are almost completely ignored by research. More holistic microbiome assessments should be conducted in the future.
6. Viruses, phages, plasmids, free (relic) DNA and various mobile genetic elements also belong to the plant microbiome; however, they are completely understudied despite indications of their importance. More studies on their implications for the assembly and functioning of the microbiome are required.
7. Most of the studies are based on amplicon sequencing of 16S (or 18S) rRNA gene fragments and the ITS region. However, such approaches only allow assessments of the community composition. More research on functions and activities occurring within the plant microbiome will be crucial for its deepening exploration. This requires more poly-phasic and inter-linked experimental and methodological approaches considering spatial and temporal scales. For studies targeting the plant microbiome, we especially suggest the combination of metagenomics/metatranscriptomics and cultivation-based approaches. This will allow an assessment of modes of (inter-)action.
8. Microbiome research provides a knowledge base that has an enormous potential to improve agricultural practices and develop microbiome-based fertilizers and pesticides. The plant microbiome is inter-connected with other organisms and ecosystems; this has to be considered especially for health issues. The development of solutions to restore and save microbial diversity for ecosystem functioning as well as the closely connected planetary health should be a central aim of microbiome researchers in the future [103].

## Electronic supplementary material

Below is the link to the electronic supplementary material.

Supplementary Material 1

Supplementary Material 2

## Data Availability

Not applicable.
