# Supplementary material for: The microbiome of cereal plants: The current state of knowledge and the potential for future applications
Source: Environ Microbiome. 2023 Mar 31;18:28. doi: 10.1186/s40793-023-00484-y (PMC10064690; doi:10.1186/s40793-023-00484-y)
Supplement: Supplementary file 1 — Supplementary Material 1 [file 40793_2023_484_MOESM1_ESM.docx]

Supplementary Information for:

**The microbiome of cereal plants: The current state of knowledge and the potential for future applications**

Kristina Michl, Gabriele Berg, and Tomislav Cernava

**Supplementary Methods**

A meta-analysis of microbial alpha diversity between the four plant types in any compartment was conducted (Fig. 4). The utilized data were extracted from 160 manuscripts depicting alpha diversity as observed ASVs/OTUs, ASV/OTU richness or Chao1 index from main text, tables, plots, or the supplementary material. The lower and upper quartiles of boxplots were extracted or the mean values with the standard deviation to build ranges. For simplicity middle values of these ranges are depicted in Fig. 4, plots showing the ranges can be found in Supplementary Figure 1 and additional information (e.g. exact values) in Supplementary Table 1.


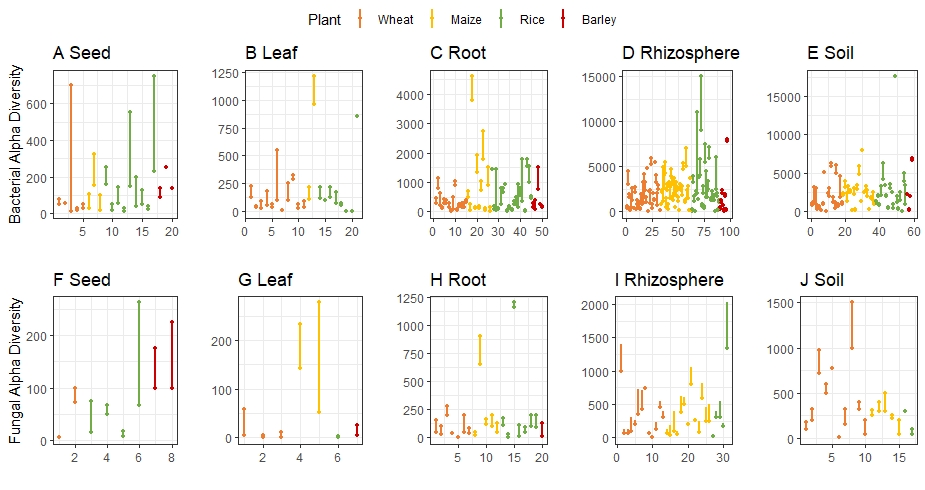


**Supplementary Figure 1. Assessment of bacterial (A-E) and fungal (F-J) diversity in the different compartments of cereals depicted as ranges.** The diversity values were extracted as chao1 index or ASV richness from the respective manuscript text or from included plots. The diversity for each compartment was found to not be significantly different between the compared cereal plants.

Supplementary References

Aguirre-von-Wobeser, Eneas; Rocha-Estrada, Jorge; Shapiro, Lori R.; La Torre, Mayra de (2018): Enrichment of Verrucomicrobia, Actinobacteria and Burkholderiales drives selection of bacterial community from soil by maize roots in a traditional milpa agroecosystem. In: *PloS one* 13 (12), e0208852.

Akimoto-Tomiyama, Chiharu (2021): Multiple endogenous seed-born bacteria recovered rice growth disruption caused by Burkholderia glumae. In: *Scientific reports* 11 (1), S. 1–12.

Akinola, Saheed Adekunle; Ayangbenro, Ayansina Segun; Babalola, Olubukola Oluranti (2021a): Metagenomic Insight into the Community Structure of Maize-Rhizosphere Bacteria as Predicted by Different Environmental Factors and Their Functioning within Plant Proximity. In: *Microorganisms* 9 (7), S. 1419.

Akinola, Saheed Adekunle; Ayangbenro, Ayansina Segun; Babalola, Olubukola Oluranti (2021b): The diverse functional genes of maize rhizosphere microbiota assessed using shotgun metagenomics. In: *Journal of the Science of Food and Agriculture* 101 (8), S. 3193–3201.

Akinola, Saheed Adekunle; Ayangbenro, Ayansina Segun; Babalola, Olubukola Oluranti (2021c): The immense functional attributes of maize rhizosphere microbiome: a shotgun sequencing approach. In: *Agriculture* 11 (2), S. 118.

Alegria Terrazas, Rodrigo; Balbirnie-Cumming, Katharin; Morris, Jenny; Hedley, Pete E.; Russell, Joanne; Paterson, Eric et al. (2020): A footprint of plant eco-geographic adaptation on the composition of the barley rhizosphere bacterial microbiota. In: *Scientific reports* 10 (1), S. 1–13.

Aliche, Ernest B.; Talsma, Warner; Munnik, Teun; Bouwmeester, Harro J. (2021): Characterization of maize root microbiome in two different soils by minimizing plant DNA contamination in metabarcoding analysis. In: *Biology and Fertility of Soils* 57 (5), S. 731–737.

Amadou, Abdoulaye; Song, Alin; Tang, Zhi-Xi; Li, Yanling; Wang, En-Zhao; Lu, Yu-Qiu et al. (2020): The effects of organic and mineral fertilization on soil enzyme activities and bacterial community in the below-and above-ground parts of wheat. In: *Agronomy* 10 (10), S. 1452.

Amina, Bouherama; Samir, Djouadi; Martinus, Schneijderberg; Ton, Bisseling; Said, Amrani (2021): Structure and composition of barley rhizospheric bacterial community and plant development cultivated with a super absorbent polymer. In: *Acta Agriculturae Scandinavica, Section B—Soil & Plant Science* 71 (6), S. 478–488.

Araujo, Ademir Sergio Ferreira de; Miranda, Ana Roberta Lima; Sousa, Ricardo Silva; Mendes, Lucas William; Antunes, Jadson Emanuel Lopes; Souza Oliveira, Louise Melo de et al. (2019a): Bacterial community associated with rhizosphere of maize and cowpea in a subsequent cultivation. In: *Applied soil ecology* 143, S. 26–34.

Araujo, Ricardo; Dunlap, Christopher; Barnett, Steve; Franco, Christopher M. M. (2019b): Decoding wheat endosphere–rhizosphere microbiomes in Rhizoctonia solani–infested soils challenged by Streptomyces biocontrol agents. In: *Frontiers in plant science*, S. 1038.

Araujo, Ricardo; Dunlap, Christopher; Franco, Christopher M. M. (2020): Analogous wheat root rhizosphere microbial successions in field and greenhouse trials in the presence of biocontrol agents Paenibacillus peoriae SP9 and Streptomyces fulvissimus FU14. In: *Molecular plant pathology* 21 (5), S. 622–635.

Azarbad, Hamed; Tremblay, Julien; Giard-Laliberte, Charlotte; Bainard, Luke D.; Yergeau, Etienne (2020): Four decades of soil water stress history together with host genotype constrain the response of the wheat microbiome to soil moisture. In: *FEMS microbiology ecology* 96 (7), fiaa098.

Baraniya, Divyashri; Nannipieri, Paolo; Kublik, Susanne; Vestergaard, Gisle; Schloter, Michael; Schöler, Anne (2018): The impact of the diurnal cycle on the microbial transcriptome in the rhizosphere of barley. In: *Microbial ecology* 75 (4), S. 830–833.

Bei, Shuikuan; Zhang, Yunlong; Li, Tengteng; Christie, Peter; Li, Xiaolin; Zhang, Junling (2018): Response of the soil microbial community to different fertilizer inputs in a wheat-maize rotation on a calcareous soil. In: *Agriculture, Ecosystems & Environment* 260, S. 58–69.

Beule, Lukas; Karlovsky, Petr (2021): Tree rows in temperate agroforestry croplands alter the composition of soil bacterial communities. In: *PloS one* 16 (2), e0246919.

Borrell, A. Navarro; Shi, Y.; Gan, Y.; Bainard, L. D.; Germida, J. J.; Hamel, C. (2017): Fungal diversity associated with pulses and its influence on the subsequent wheat crop in the Canadian prairies. In: *Plant and Soil* 414 (1), S. 13–31.

Breidenbach, Björn; Pump, Judith; Dumont, Marc G. (2016): Microbial community structure in the rhizosphere of rice plants. In: *Frontiers in Microbiology* 6, S. 1537.

Breitkreuz, Claudia; Herzig, Laura; Buscot, François; Reitz, Thomas; Tarkka, Mika (2021): Interactions between soil properties, agricultural management and cultivar type drive structural and functional adaptations of the wheat rhizosphere microbiome to drought. In: *Environmental Microbiology* 23 (10), S. 5866–5882.

Brisson, Vanessa L.; Schmidt, Jennifer E.; Northen, Trent R.; Vogel, John P.; Gaudin, Amélie (2019): Impacts of maize domestication and breeding on rhizosphere microbial community recruitment from a nutrient depleted agricultural soil. In: *Scientific reports* 9 (1), S. 1–14.

Cadot, Selma; Guan, Hang; Bigalke, Moritz; Walser, Jean-Claude; Jander, Georg; Erb, Matthias et al. (2021): Specific and conserved patterns of microbiota-structuring by maize benzoxazinoids in the field. In: *Microbiome* 9 (1), S. 1–19.

Cardinale, Massimiliano; Suarez, Christian; Steffens, Diedrich; Ratering, Stefan; Schnell, Sylvia (2019): Effect of different soil phosphate sources on the active bacterial microbiota is greater in the rhizosphere than in the endorhiza of barley (Hordeum vulgare L.). In: *Microbial ecology* 77 (3), S. 689–700.

Chang, Jingjing; Shi, Shaohua; Tian, Lei; Leite, Marcio F. A.; Chang, Chunling; Ji, Li et al. (2021a): Self-crossing leads to weak co-variation of the bacterial and fungal communities in the rice rhizosphere. In: *Microorganisms* 9 (1), S. 175.

Chang, Jingjing; Sun, Yu; Tian, Lei; Ji, Li; Luo, Shasha; Nasir, Fahad et al. (2021b): The structure of rhizosphere fungal communities of wild and domesticated rice: Changes in diversity and co-occurrence patterns. In: *Frontiers in Microbiology* 12, S. 45.

Chaudhary, Parul; Khati, Priyanka; Chaudhary, Anuj; Maithani, Damini; Kumar, Govind; Sharma, Anita (2021): Cultivable and metagenomic approach to study the combined impact of nanogypsum and Pseudomonas taiwanensis on maize plant health and its rhizospheric microbiome. In: *PloS one* 16 (4), e0250574.

Chen, Qing-Lin; An, Xin-Li; Zheng, Bang-Xiao; Ma, Yi-Bing; Su, Jian-Qiang (2018): Long-term organic fertilization increased antibiotic resistome in phyllosphere of maize. In: *Science of The Total Environment* 645, S. 1230–1237.

Cobo-Diaz, Jose F.; Legrand, Fabienne; Le Floch, Gaetan; Picot, Adeline (2021): Influence of Maize Residues in Shaping Soil Microbiota and Fusarium spp. Communities. In: *Microbial ecology*, S. 1–12.

Cordero Elvia, Jorge; Freitas, J. Renato de; Germida, James J. (2021): Bacterial Microbiomes Associated with the Rhizosphere, Root Interior, and Aboveground Plant Organs of Wheat and Canola at Different Growth Stages. In: *Phytobiomes Journal* 5 (4), S. 442–451.

Correa-Galeote, David; Bedmar, Eulogio J.; Arone, Gregorio J. (2018): Maize endophytic bacterial diversity as affected by soil cultivation history. In: *Frontiers in Microbiology* 9, S. 484.

Correa-Galeote, David; Bedmar, Eulogio J.; Fernández-González, Antonio J.; Fernández-López, Manuel; Arone, Gregorio J. (2016): Bacterial communities in the rhizosphere of amilaceous maize (Zea mays L.) as assessed by pyrosequencing. In: *Frontiers in plant science* 7, S. 1016.

Cotton, T. E.; Pétriacq, Pierre; Cameron, Duncan D.; Meselmani, Moaed Al; Schwarzenbacher, Roland; Rolfe, Stephen A.; Ton, Jurriaan (2019): Metabolic regulation of the maize rhizobiome by benzoxazinoids. In: *The ISME journal* 13 (7), S. 1647–1658.

Cui, Erping; Fan, Xiangyang; Li, Zhongyang; Liu, Yuan; Neal, Andrew L.; Hu, Chao; Gao, Feng (2019): Variations in soil and plant-microbiome composition with different quality irrigation waters and biochar supplementation. In: *Applied soil ecology* 142, S. 99–109.

Dai, Jun; Tang, Zhu; Jiang, Nan; Kopittke, Peter M.; Zhao, Fang-Jie; Wang, Peng (2020a): Increased arsenic mobilization in the rice rhizosphere is mediated by iron-reducing bacteria. In: *Environmental Pollution* 263, S. 114561.

Dai, Yumei; Yang, Feng; Zhang, Lingli; Xu, Zhibin; Fan, Xiaoli; Tian, Yongqiang; Wang, Tao (2020b): Wheat‐associated microbiota and their correlation with stripe rust reaction. In: *Journal of Applied Microbiology* 128 (2), S. 544–555.

Dal Cortivo, Cristian; Ferrari, Manuel; Visioli, Giovanna; Lauro, Marta; Fornasier, Flavio; Barion, Giuseppe et al. (2020): Effects of seed-applied biofertilizers on rhizosphere biodiversity and growth of common wheat (Triticum aestivum L.) in the field. In: *Frontiers in plant science* 11, S. 72.

Das, Suvendu; Gwon, Hyo Suk; Khan, Muhammad Israr; van Nostrand, Joy D.; Alam, Muhammad Ashraful; Kim, Pil Joo (2019): Taxonomic and functional responses of soil microbial communities to slag-based fertilizer amendment in rice cropping systems. In: *Environment international* 127, S. 531–539.

Edwards, Joseph; Johnson, Cameron; Santos-Medellín, Christian; Lurie, Eugene; Podishetty, Natraj Kumar; Bhatnagar, Srijak et al. (2015): Structure, variation, and assembly of the root-associated microbiomes of rice. In: *Proceedings of the National Academy of Sciences* 112 (8), E911-E920.

Edwards, Joseph A.; Santos-Medellín, Christian M.; Liechty, Zachary S.; Nguyen, Bao; Lurie, Eugene; Eason, Shane et al. (2018): Compositional shifts in root-associated bacterial and archaeal microbiota track the plant life cycle in field-grown rice. In: *PLoS biology* 16 (2), e2003862.

Emmett, Bryan D.; Buckley, Daniel H.; Smith, Margaret E.; Drinkwater, Laurie E. (2018): Eighty years of maize breeding alters plant nitrogen acquisition but not rhizosphere bacterial community composition. In: *Plant and Soil* 431 (1), S. 53–69.

Emmett, Bryan D.; Youngblut, Nicholas D.; Buckley, Daniel H.; Drinkwater, Laurie E. (2017): Plant phylogeny and life history shape rhizosphere bacterial microbiome of summer annuals in an agricultural field. In: *Frontiers in Microbiology* 8, S. 2414.

Enebe, Matthew Chekwube; Babalola, Olubukola Oluranti (2020): Effects of inorganic and organic treatments on the microbial community of maize rhizosphere by a shotgun metagenomics approach. In: *Annals of Microbiology* 70 (1), S. 1–10.

Eyre, Alexander W.; Wang, Mengying; Oh, Yeonyee; Dean, Ralph A. (2019): Identification and characterization of the core rice seed microbiome. In: *Phytobiomes Journal* 3 (2), S. 148–157.

Fabiańska, Izabela; Pesch, Lina; Koebke, Eva; Gerlach, Nina; Bucher, Marcel (2020): Neighboring plants divergently modulate effects of loss-of-function in maize mycorrhizal phosphate uptake on host physiology and root fungal microbiota. In: *PloS one* 15 (6), e0232633.

Fadiji, Ayomide Emmanuel; Ayangbenro, Ayansina Segun; Babalola, Olubukola Oluranti (2021a): Unveiling the putative functional genes present in root-associated endophytic microbiome from maize plant using the shotgun approach. In: *Journal of Applied Genetics* 62 (2), S. 339–351.

Fadiji, Ayomide Emmanuel; Kanu, Jerry Onyemaechi; Babalola, Olubukola Oluranti (2021b): Impact of cropping systems on the functional diversity of rhizosphere microbial communities associated with maize plant: a shotgun approach. In: *Archives of Microbiology* 203 (6), S. 3605–3613.

Fadiji, Ayomide Emmanuel; Kanu, Jerry Onyemaechi; Babalola, Olubukola Oluranti (2021c): Metagenomic profiling of rhizosphere microbial community structure and diversity associated with maize plant as affected by cropping systems. In: *International Microbiology* 24 (3), S. 325–335.

Fan, Kunkun; Cardona, Cesar; Li, Yuntao; Shi, Y. U.; Xiang, Xingjia; Shen, Congcong et al. (2017): Rhizosphere-associated bacterial network structure and spatial distribution differ significantly from bulk soil in wheat crop fields. In: *Soil Biology and Biochemistry* 113, S. 275–284.

Favela, Alonso; O Bohn, Martin; D Kent, Angela (2021): Maize germplasm chronosequence shows crop breeding history impacts recruitment of the rhizosphere microbiome. In: *The ISME journal* 15 (8), S. 2454–2464.

Feng, Jiayin; Franks, Ashley E.; Lu, Zhijiang; Xu, Jianming; He, Yan (2021): Assembly and variation of root-associated microbiota of rice during their vegetative growth phase with and without lindane pollutant. In: *Soil Ecology Letters* 3 (3), S. 207–219.

Feng, Jiayin; Zhu, Yanjie; Shentu, Jue; Lu, Zhijiang; He, Yan; Xu, Jianming (2020): Pollution adaptive responses of root-associated microbiomes induced the promoted but different attenuation of soil residual lindane: Differences between maize and soybean. In: *Science of The Total Environment* 732, S. 139170.

Fernández-Baca, Cristina P.; Rivers, Adam R.; Maul, Jude E.; Kim, Woojae; Poudel, Ravin; McClung, Anna M. et al. (2021): Rice Plant–Soil Microbiome Interactions Driven by Root and Shoot Biomass. In: *Diversity* 13 (3), S. 125.

Figueiredo dos Santos, Lidiane; Fernandes Souta, Julie; Paula Soares, Cleiton de; Da Oliveira Rocha, Letícia; Luiza Carvalho Santos, Maria; Grativol, Clicia et al. (2021): Insights into the structure and role of seed-borne bacteriome during maize germination. In: *FEMS microbiology ecology* 97 (4), fiab024.

Frindte, Katharina; Zoche, Sarah A.; Knief, Claudia (2020): Development of a Distinct Microbial Community Upon First Season Crop Change in Soils of Long-Term Managed Maize and Rice Fields. In: *Frontiers in Microbiology*, S. 2749.

Gałązka, Anna; Grządziel, Jarosław (2018): Fungal genetics and functional diversity of microbial communities in the soil under long-term monoculture of maize using different cultivation techniques. In: *Frontiers in Microbiology* 9, S. 76.

Gdanetz, Kristi; Trail, Frances (2017): The wheat microbiome under four management strategies, and potential for endophytes in disease protection. In: *Phytobiomes Journal* 1 (3), S. 158–168.

Gebauer, Lucie; Bouffaud, Marie-Lara; Ganther, Minh; Yim, Bunlong; Vetterlein, Doris; Smalla, Kornelia et al. (2021): Soil texture, sampling depth and root hairs shape the structure of ACC deaminase bacterial community composition in maize rhizosphere. In: *Frontiers in Microbiology*, S. 128.

Giard-Laliberté, Charlotte; Azarbad, Hamed; Tremblay, Julien; Bainard, Luke; Yergeau, Étienne (2019): A water stress-adapted inoculum affects rhizosphere fungi, but not bacteria nor wheat. In: *FEMS microbiology ecology* 95 (7), fiz080.

Gomes, Eliane A.; Lana, Ubiraci G. P.; Quensen, John F.; Sousa, Sylvia M. de; Oliveira, Christiane A.; Guo, Jiarong et al. (2018): Root-associated microbiome of maize genotypes with contrasting phosphorus use efficiency. In: *Phytobiomes Journal* 2 (3), S. 129–137.

Gqozo, Magalane Pheladi; Bill, Malick; Siyoum, Nazareth; Labuschagne, Nico; Korsten, Lise (2020): Fungal diversity and community composition of wheat rhizosphere and non-rhizosphere soils from three different agricultural production regions of South Africa. In: *Applied soil ecology* 151, S. 103543.

Granzow, Sandra; Kaiser, Kristin; Wemheuer, Bernd; Pfeiffer, Birgit; Daniel, Rolf; Vidal, Stefan; Wemheuer, Franziska (2017): The effects of cropping regimes on fungal and bacterial communities of wheat and faba bean in a greenhouse pot experiment differ between plant species and compartment. In: *Frontiers in Microbiology* 8, S. 902.

Guimarães, Rafaela Araújo; Pherez-Perrony, Paul Esteban; Müller, Henry; Berg, Gabriele; Medeiros, Flávio Henrique Vasconcelos; Cernava, Tomislav (2020): Microbiome-guided evaluation of Bacillus subtilis BIOUFLA2 application to reduce mycotoxins in maize kernels. In: *Biological control* 150, S. 104370.

Guo, Junjie; Ling, Ning; Li, Yong; Li, Kaisong; Ning, Huiling; Shen, Qirong et al. (2021): Seed‐borne, endospheric and rhizospheric core microbiota as predictors of plant functional traits across rice cultivars are dominated by deterministic processes. In: *New Phytologist* 230 (5), S. 2047–2060.

Hamel, Chantal; Gan, Yantai; Sokolski, Serge; Bainard, Luke D. (2018): High frequency cropping of pulses modifies soil nitrogen level and the rhizosphere bacterial microbiome in 4-year rotation systems of the semiarid prairie. In: *Applied soil ecology* 126, S. 47–56.

Hao, Lijun; Zhang, Zhechao; Hao, Baihui; Diao, Fengwei; Zhang, Jingxia; Bao, Zhihua; Guo, Wei (2021): Arbuscular mycorrhizal fungi alter microbiome structure of rhizosphere soil to enhance maize tolerance to La. In: *Ecotoxicology and Environmental Safety* 212, S. 111996.

Hartmann, Martin; Frey, Beat; Mayer, Jochen; Mäder, Paul; Widmer, Franco (2015): Distinct soil microbial diversity under long-term organic and conventional farming. In: *The ISME journal* 9 (5), S. 1177–1194.

Hayden, Helen L.; Savin, Keith W.; Wadeson, Jenny; Gupta, Vadakattu VSR; Mele, Pauline M. (2018): Comparative metatranscriptomics of wheat rhizosphere microbiomes in disease suppressive and non-suppressive soils for Rhizoctonia solani AG8. In: *Frontiers in Microbiology* 9, S. 859.

He, Anle; Sun, Jianan; Wang, Xinhua; Zou, Liwen; Fu, Bo; Chen, Jie (2019): Reprogrammed endophytic microbial community in maize stalk induced by Trichoderma asperellum biocontrol agent against Fusarium diseases and mycotoxin accumulation. In: *Fungal biology* 123 (6), S. 448–455.

Hernández, Marcela; Dumont, Marc G.; Yuan, Quan; Conrad, Ralf (2015): Different bacterial populations associated with the roots and rhizosphere of rice incorporate plant-derived carbon. In: *Applied and environmental microbiology* 81 (6), S. 2244–2253.

Higo, Masao; Tatewaki, Yuya; Iida, Karen; Yokota, Kana; Isobe, Katsunori (2020): Amplicon sequencing analysis of arbuscular mycorrhizal fungal communities colonizing maize roots in different cover cropping and tillage systems. In: *Scientific reports* 10 (1), S. 1–13.

Hou, Dandi; Wang, Runze; Gao, Xiaoyu; Wang, Kai; Lin, Zhi; Ge, Jun et al. (2018): Cultivar-specific response of bacterial community to cadmium contamination in the rhizosphere of rice (Oryza sativa L.). In: *Environmental Pollution* 241, S. 63–73.

Hou, Jinyu; Wu, Longhua; Liu, Wuxing; Ge, Yanyan; Mu, Tingting; Zhou, Tong et al. (2020): Biogeography and diversity patterns of abundant and rare bacterial communities in rice paddy soils across China. In: *Science of The Total Environment* 730, S. 139116.

Huang, Ruilin; Ding, Jixian; Guo, Yuwei; Sun, Bo; Liang, Yuting (2022): Habitat determines the relationships among bacteria, resistance genes and mobile genetic elements in the soil–plant system. In: *European Journal of Soil Science* 73 (1), e13132.

Huang, Yali; Kuang, Zaoyuan; Wang, Wenfeng; Cao, Lixiang (2016): Exploring potential bacterial and fungal biocontrol agents transmitted from seeds to sprouts of wheat. In: *Biological control* 98, S. 27–33.

Hünninghaus, Maike; Dibbern, Dörte; Kramer, Susanne; Koller, Robert; Pausch, Johanna; Schloter-Hai, Brigitte et al. (2019): Disentangling carbon flow across microbial kingdoms in the rhizosphere of maize. In: *Soil Biology and Biochemistry* 134, S. 122–130.

Hyun, Hye Rim; Yoon, Hakwon; Lyou, Eun Sun; Kim, Jin Ju; Kwon, Sae Yun; Lee, Tae Kwon (2021): Short-Term Legacy Effects of Mercury Contamination on Plant Growth and nifH-Harboring Microbial Community in Rice Paddy Soil. In: *Microbial ecology* 82 (4), S. 932–941.

Igor, Kazartsev; Tatiana, Gagkaeva; Olga, Gavrilova; Philipp, Gannibal (2020): Fungal microbiome of barley grain revealed by NGS and mycological analysis. In: *Foods and Raw materials* 8 (2), S. 286–297.

Ikeda, Seishi; Sasaki, Kazuhiro; Okubo, Takashi; Yamashita, Akifumu; Terasawa, Kimihiro; Bao, Zhihua et al. (2014): Low nitrogen fertilization adapts rice root microbiome to low nutrient environment by changing biogeochemical functions. In: *Microbes and environments*, ME13110.

Ishaq, Suzanne L.; Seipel, Tim; Yeoman, Carl J.; Menalled, Fabian D. (2020): Soil bacterial communities of wheat vary across the growing season and among dryland farming systems. In: *Geoderma* 358, S. 113989.

Jeewani, Peduruhewa H.; Chen, Lin; van Zwieten, Lukas; Shen, Congcong; Guggenberger, Georg; Luo, Yu; Xu, Jianming (2020): Shifts in the bacterial community along with root-associated compartments of maize as affected by goethite. In: *Biology and Fertility of Soils* 56 (8), S. 1201–1210.

Jha, Prabhat N.; Gomaa, Abu-Bakr; Yanni, Youssef G.; El-Saadany, Abd-Elgawad Y.; Stedtfeld, Tiffany M.; Stedtfeld, Robert D. et al. (2020): Alterations in the endophyte-enriched root-associated microbiome of rice receiving growth-promoting treatments of urea fertilizer and Rhizobium biofertilizer. In: *Microbial ecology* 79 (2), S. 367–382.

Jiménez‐Bueno, N. G.; Valenzuela‐Encinas, C.; Marsch, R.; Ortiz‐Gutiérrez, D.; Verhulst, N.; Govaerts, B. et al. (2016): Bacterial indicator taxa in soils under different long‐term agricultural management. In: *Journal of Applied Microbiology* 120 (4), S. 921–933.

Johnston-Monje, David; Lundberg, Derek S.; Lazarovits, George; Reis, Veronica M.; Raizada, Manish N. (2016): Bacterial populations in juvenile maize rhizospheres originate from both seed and soil. In: *Plant and Soil* 405 (1), S. 337–355.

Kanasugi, Makoto; Sarkodee-Addo, Elsie; Ansong Omari, Richard; Mohammad Golam Dastogeer, Khondoker; Fujii, Yoshiharu; Oppong Abebrese, Samuel et al. (2020): Exploring Rice Root Microbiome; the variation, specialization and interaction of bacteria and fungi in six tropic savanna regions in Ghana. In: *Sustainability* 12 (14), S. 5835.

Karlsson, Ida; Friberg, Hanna; Steinberg, Christian; Persson, Paula (2014): Fungicide effects on fungal community composition in the wheat phyllosphere. In: *PloS one* 9 (11), e111786.

Kavamura, Vanessa N.; Robinson, Rebekah J.; Hughes, David; Clark, Ian; Rossmann, Maike; Melo, Itamar Soares de et al. (2020): Wheat dwarfing influences selection of the rhizosphere microbiome. In: *Scientific reports* 10 (1), S. 1–11.

Kavamura, Vanessa Nessner; Robinson, Rebekah J.; Hayat, Rifat; Clark, Ian M.; Hughes, David; Rossmann, Maike et al. (2019): Land management and microbial seed load effect on rhizosphere and endosphere bacterial community assembly in wheat. In: *Frontiers in Microbiology*, S. 2625.

Kawasaki, Akitomo; Dennis, Paul G.; Forstner, Christian; Raghavendra, Anil K. H.; Richardson, Alan E.; Watt, Michelle et al. (2021): The microbiomes on the roots of wheat (Triticum aestivum L.) and rice (Oryza sativa L.) exhibit significant differences in structure between root types and along root axes. In: *Functional Plant Biology* 48 (9), S. 871–888.

Kim, Hyun; Lee, Kiseok Keith; Jeon, Jongbum; Harris, William Anthony; Lee, Yong-Hwan (2020): Domestication of Oryza species eco-evolutionarily shapes bacterial and fungal communities in rice seed. In: *Microbiome* 8 (1), S. 1–17.

Knief, Claudia; Delmotte, Nathanaël; Chaffron, Samuel; Stark, Manuel; Innerebner, Gerd; Wassmann, Reiner et al. (2012): Metaproteogenomic analysis of microbial communities in the phyllosphere and rhizosphere of rice. In: *The ISME journal* 6 (7), S. 1378–1390.

Kong, Xiao; Han, Zhenfei; Tai, Xin; Jin, Decai; Ai, Sen; Zheng, Xiaoxu; Bai, Zhihui (2020): Maize (Zea mays L. Sp.) varieties significantly influence bacterial and fungal community in bulk soil, rhizosphere soil and phyllosphere. In: *FEMS microbiology ecology* 96 (3), fiaa020.

Kumar, Vinod; AlMomin, Sabah; Al-Aqeel, Hamed; Al-Salameen, Fadila; Nair, Sindhu; Shajan, Anisha (2018): Metagenomic analysis of rhizosphere microflora of oil-contaminated soil planted with barley and alfalfa. In: *PloS one* 13 (8), e0202127.

Kusstatscher, Peter; Wicaksono, Wisnu Adi; Thenappan, Dhivya P.; Adam, Eveline; Müller, Henry; Berg, Gabriele (2020): Microbiome management by biological and chemical treatments in maize is linked to plant health. In: *Microorganisms* 8 (10), S. 1506.

Kuźniar, Agnieszka; Włodarczyk, Kinga; Grządziel, Jarosław; Goraj, Weronika; Gałązka, Anna; Wolińska, Agnieszka (2020a): Culture-independent analysis of an endophytic core microbiome in two species of wheat: Triticum aestivum L.(cv.‘Hondia’) and the first report of microbiota in Triticum spelta L.(cv.‘Rokosz’). In: *Systematic and Applied Microbiology* 43 (1), S. 126025.

Kuźniar, Agnieszka; Włodarczyk, Kinga; Grządziel, Jarosław; Woźniak, Małgorzata; Furtak, Karolina; Gałązka, Anna et al. (2020b): New insight into the composition of wheat seed microbiota. In: *International journal of molecular sciences* 21 (13), S. 4634.

L. Neal, Andrew; McLaren, Timothy; Lourenço Campolino, Mariana; Hughes, David; Marcos Coelho, Antônio; Gomes de Paula Lana, Ubiraci et al. (2021): Crop type exerts greater influence upon rhizosphere phosphohydrolase gene abundance and phylogenetic diversity than phosphorus fertilization. In: *FEMS microbiology ecology* 97 (4), fiab033.

Lang, Ming; Bei, Shuikuan; Li, Xia; Kuyper, Thomas W.; Zhang, Junling (2019): Rhizoplane bacteria and plant species co-determine phosphorus-mediated microbial legacy effect. In: *Frontiers in Microbiology*, S. 2856.

Lang, Ming; Christie, Peter; Zhang, Junling; Li, Xiaolin (2018): Long-term phosphorus application to a maize monoculture influences the soil microbial community and its feedback effects on maize seedling biomass. In: *Applied soil ecology* 128, S. 12–22.

Lang, Ming; Zou, Wenxin; Chen, Xiuxiu; Zou, Chunqin; Zhang, Wei; Deng, Yan et al. (2021): Soil microbial composition and phod gene abundance are sensitive to phosphorus level in a long-term wheat-maize crop system. In: *Frontiers in Microbiology*, S. 3547.

Latif, Sadia; Bibi, Sameeda; Kouser, Rabia; Fatimah, Hina; Farooq, Saba; Naseer, Samar; Kousar, Rizwana (2020): Characterization of bacterial community structure in the rhizosphere of Triticum aestivum L. In: *Genomics* 112 (6), S. 4760–4768.

Latini, Arianna; Bacci, Giovanni; Teodoro, Manuel; Gattia, Daniele Mirabile; Bevivino, Annamaria; Trakal, Lukáš (2019): The impact of soil-applied biochars from different vegetal feedstocks on durum wheat plant performance and rhizospheric bacterial microbiota in low metal-contaminated soil. In: *Frontiers in Microbiology* 10, S. 2694.

Li, Baoqin; Xu, Rui; Sun, Xiaoxu; Han, Feng; Xiao, Enzong; Chen, Lei et al. (2021a): Microbiome–environment interactions in antimony-contaminated rice paddies and the correlation of core microbiome with arsenic and antimony contamination. In: *Chemosphere* 263, S. 128227.

Li, Hong-Yi; Wang, Hang; Wang, Hai-Tao; Xin, Pei-Yong; Xu, Xin-Hua; Ma, Yun et al. (2018a): The chemodiversity of paddy soil dissolved organic matter correlates with microbial community at continental scales. In: *Microbiome* 6 (1), S. 1–16.

Li, Xiangzhen; Rui, Junpeng; Mao, Yuejian; Yannarell, Anthony; Mackie, Roderick (2014): Dynamics of the bacterial community structure in the rhizosphere of a maize cultivar. In: *Soil Biology and Biochemistry* 68, S. 392–401.

Li, Yaofa; An, Jingjie; Dang, Zhihong; Lv, Haiying; Pan, Wenliang; Gao, Zhanlin (2018b): Treating wheat seeds with neonicotinoid insecticides does not harm the rhizosphere microbial community. In: *PloS one* 13 (12), e0205200.

Li, Yongbin; Wang, Minyang; Chen, Sanfeng (2021b): Application of N2-fixing Paenibacillus triticisoli BJ-18 changes the compositions and functions of the bacterial, diazotrophic, and fungal microbiomes in the rhizosphere and root/shoot endosphere of wheat under field conditions. In: *Biology and Fertility of Soils* 57 (3), S. 347–362.

Li, Yunliang; Laterrière, Mario; Lay, Chih-Ying; Klabi, Rim; Masse, Jacynthe; St-Arnaud, Marc et al. (2021c): Effects of arbuscular mycorrhizal fungi inoculation and crop sequence on root-associated microbiome, crop productivity and nutrient uptake in wheat-based and flax-based cropping systems. In: *Applied soil ecology* 168, S. 104136.

Li, Yüze; Li, Tong; Zhao, Deqiang; Wang, Ziting; Liao, Yuncheng (2021d): Different tillage practices change assembly, composition, and co-occurrence patterns of wheat rhizosphere diazotrophs. In: *Science of The Total Environment* 767, S. 144252.

Lian, Tengxiang; Huang, Yingyong; Xie, Xianan; Huo, Xing; Shahid, Muhammad Qasim; Tian, Lei et al. (2020): Rice SST variation shapes the rhizosphere bacterial community, conferring tolerance to salt stress through regulating soil metabolites. In: *MSystems* 5 (6), e00721-20.

Lin, CHEN; Xiuli, X. I.N.; Zhang, Jiabao; Redmile-Gordon, Marc; Guangsen, N. I.E.; Qingyun, WANG (2019): Soil characteristics overwhelm cultivar effects on the structure and assembly of root-associated microbiomes of modern maize. In: *Pedosphere* 29 (3), S. 360–373.

Liu, Hongwei; Carvalhais, Lilia C.; Schenk, Peer M.; Dennis, Paul G. (2017a): Effects of jasmonic acid signalling on the wheat microbiome differ between body sites. In: *Scientific reports* 7 (1), S. 1–8.

Liu, Hongwei; Carvalhais, Lilia C.; Schenk, Peer M.; Dennis, Paul G. (2018): Activation of the salicylic acid signalling pathway in wheat had no significant short-term impact on the diversity of root-associated microbiomes. In: *Pedobiologia* 70, S. 6–11.

Liu, Wenbo; Ling, Ning; Guo, Junjie; Ruan, Yang; Zhu, Chen; Shen, Qirong; Guo, Shiwei (2020a): Legacy effects of 8-year nitrogen inputs on bacterial assemblage in wheat rhizosphere. In: *Biology and Fertility of Soils* 56 (5), S. 583–596.

Liu, Yang; Wang, Ronghuan; Li, Yinhu; Cao, Yanhua; Chen, Chuanyong; Qiu, Chuangzhao et al. (2017b): High-throughput sequencing-based analysis of the composition and diversity of endophytic bacterial community in seeds of “Beijing” hybrid maize planted in China. In: *Plant Growth Regulation* 81 (2), S. 317–324.

Liu, Yang; Yan, Hai; Zhang, Xiaoxia; Zhang, Ruyang; Li, Miao; Xu, Tianjun et al. (2020b): Investigating the endophytic bacterial diversity and community structures in seeds of genetically related maize (Zea mays L.) genotypes. In: *3 Biotech* 10 (1), S. 1–10.

Liu, Z. D.; Li, L.; Zhuo, G.; Xue, B. (2019): Characterizing structure and potential function of bacterial and fungal root microbiota in hulless barley cultivars. In: *Journal of Soil Science and Plant Nutrition* 19 (2), S. 420–429.

Lv, Zhiyao; Dai, Rui; Xu, Haoran; Liu, Yongxin; Bai, Bo; Meng, Ying et al. (2021): The rice histone methylation regulates hub species of the root microbiota. In: *Journal of Genetics and Genomics* 48 (9), S. 836–843.

Ma, Zheng; Yi, Zhihao; Bayar, Kaanuru; Fu, Yuming; Liu, Hong (2021): Community dynamics in rhizosphere microorganisms at different development stages of wheat growing in confined isolation environments. In: *Applied Microbiology and Biotechnology* 105 (9), S. 3843–3857.

Mahoney, Aaron K.; Yin, Chuntao; Hulbert, Scot H. (2017): Community structure, species variation, and potential functions of rhizosphere-associated bacteria of different winter wheat (Triticum aestivum) cultivars. In: *Frontiers in plant science* 8, S. 132.

Majumdar, Rajtilak; Kandel, Shyam L.; Cary, Jeffrey W.; Rajasekaran, Kanniah (2021): Changes in Bacterial Endophyte Community Following Aspergillusflavus Infection in Resistant and Susceptible Maize Kernels. In: *International journal of molecular sciences* 22 (7), S. 3747.

Mashiane, Ramadimetja A.; Ezeokoli, Obinna T.; Adeleke, Rasheed A.; Bezuidenhout, Cornelius C. (2017): Metagenomic analyses of bacterial endophytes associated with the phyllosphere of a Bt maize cultivar and its isogenic parental line from South Africa. In: *World Journal of Microbiology and Biotechnology* 33 (4), S. 1–12.

Masson, Anne-Sophie; Ho Bich, Hai; Simonin, Marie; Nguyen Thi, Hue; Czernic, Pierre; Moulin, Lionel; Bellafiore, Stéphane (2020): Deep modifications of the microbiome of rice roots infected by the parasitic nematode Meloidogyne graminicola in highly infested fields in Vietnam. In: *FEMS microbiology ecology* 96 (7), fiaa099.

Mauchline, T. H.; Chedom‐Fotso, D.; Chandra, G.; Samuels, T.; Greenaway, N.; Backhaus, A. et al. (2015): An analysis of P seudomonas genomic diversity in take‐all infected wheat fields reveals the lasting impact of wheat cultivars on the soil microbiota. In: *Environmental Microbiology* 17 (11), S. 4764–4778.

Mavrodi, Dmitri V.; Mavrodi, Olga V.; Elbourne, Liam D. H.; Tetu, Sasha; Bonsall, Robert F.; Parejko, James et al. (2018): Long-term irrigation affects the dynamics and activity of the wheat rhizosphere microbiome. In: *Frontiers in plant science* 9, S. 345.

Megyes, Melinda; Borsodi, Andrea K.; Árendás, Tamás; Márialigeti, Károly (2021): Variations in the diversity of soil bacterial and archaeal communities in response to different long-term fertilization regimes in maize fields. In: *Applied soil ecology* 168, S. 104120.

Meier, Michael A.; Lopez-Guerrero, Martha G.; Guo, Ming; Schmer, Marty R.; Herr, Joshua R.; Schnable, James C. et al. (2021): Rhizosphere Microbiomes in a Historical Maize-Soybean Rotation System Respond to Host Species and Nitrogen Fertilization at the Genus and Subgenus Levels. In: *Applied and environmental microbiology* 87 (12), e03132-20.

Meng, Lulu; Sun, Tong; Li, Mengyao; Saleem, Muhammad; Zhang, Qingming; Wang, Caixia (2019): Soil-applied biochar increases microbial diversity and wheat plant performance under herbicide fomesafen stress. In: *Ecotoxicology and Environmental Safety* 171, S. 75–83.

Miller, Hannah; Dias, Kandis; Hare, Hannah; Borton, Mikayla A.; Blotevogel, Jens; Danforth, Cloelle et al. (2020): Reusing oil and gas produced water for agricultural irrigation: Effects on soil health and the soil microbiome. In: *Science of The Total Environment* 722, S. 137888.

Mitter, Eduardo K.; Freitas, J. Renato de; Germida, James J. (2017): Bacterial root microbiome of plants growing in oil sands reclamation covers. In: *Frontiers in Microbiology* 8, S. 849.

Moronta-Barrios, Felix; Gionechetti, Fabrizia; Pallavicini, Alberto; Marys, Edgloris; Venturi, Vittorio (2018): Bacterial microbiota of rice roots: 16S-based taxonomic profiling of endophytic and rhizospheric diversity, endophytes isolation and simplified endophytic community. In: *Microorganisms* 6 (1), S. 14.

Murphy, Katherine M.; Edwards, Joseph; Louie, Katherine B.; Bowen, Benjamin P.; Sundaresan, Venkatesan; Northen, Trent R.; Zerbe, Philipp (2021): Bioactive diterpenoids impact the composition of the root-associated microbiome in maize (Zea mays). In: *Scientific reports* 11 (1), S. 1–13.

Mwakilili, Aneth David; Mwaikono, Kilaza Samson; Herrera, Sebastian Larsson; Midega, Charles A. O.; Magingo, Francis; Alsanius, Beatrix et al. (2021): Long-term maize-Desmodium intercropping shifts structure and composition of soil microbiome with stronger impact on fungal communities. In: *Plant and Soil* 467 (1), S. 437–450.

Nan, Qiong; Wang, Cheng; Wang, Hao; Yi, Qianqian; Liang, Biqing; Xu, Jun; Wu, Weixiang (2020): Biochar drives microbially-mediated rice production by increasing soil carbon. In: *Journal of Hazardous Materials* 387, S. 121680.

Nasir, Fahad; Shi, Shaohua; Tian, Lei; Chang, Chunling; Ma, Lina; Li, Xiujun et al. (2019): Strigolactones shape the rhizomicrobiome in rice (Oryza sativa). In: *Plant Science* 286, S. 118–133.

Niu, Ben; Paulson, Joseph Nathaniel; Zheng, Xiaoqi; Kolter, Roberto (2017): Simplified and representative bacterial community of maize roots. In: *Proceedings of the National Academy of Sciences* 114 (12), E2450-E2459.

Ofek, Maya; Voronov‐Goldman, Milana; Hadar, Yitzhak; Minz, Dror (2014): Host signature effect on plant root‐associated microbiomes revealed through analyses of resident vs. active communities. In: *Environmental Microbiology* 16 (7), S. 2157–2167.

Ofek-Lalzar, Maya; Sela, Noa; Goldman-Voronov, Milana; Green, Stefan J.; Hadar, Yitzhak; Minz, Dror (2014): Niche and host-associated functional signatures of the root surface microbiome. In: *Nature communications* 5 (1), S. 1–9.

Okubo, Takashi; Ikeda, Seishi; Sasaki, Kazuhiro; Ohshima, Kenshiro; Hattori, Masahira; Sato, Tadashi; Minamisawa, Kiwamu (2014): Phylogeny and functions of bacterial communities associated with field-grown rice shoots. In: *Microbes and environments* 29 (3), S. 329–332.

Özkurt, Ezgi; Hassani, M. Amine; Sesiz, Uğur; Künzel, Sven; Dagan, Tal; Özkan, Hakan; Stukenbrock, Eva H. (2020): Seed-derived microbial colonization of wild emmer and domesticated bread wheat (Triticum dicoccoides and T. aestivum) seedlings shows pronounced differences in overall diversity and composition. In: *MBio* 11 (6), e02637-20.

Pagé, Antoine P.; Tremblay, Julien; Masson, Luke; Greer, Charles W. (2019): Nitrogen-and phosphorus-starved Triticum aestivum show distinct belowground microbiome profiles. In: *PloS one* 14 (2), e0210538.

Pang, Zhiqiang; Xu, Peng; Yu, Diqiu (2020): Environmental adaptation of the root microbiome in two rice ecotypes. In: *Microbiological Research* 241, S. 126588.

Parizadeh, Mona; Mimee, Benjamin; Kembel, Steven W. (2021): Neonicotinoid Seed Treatments Have Significant Non-target Effects on Phyllosphere and Soil Bacterial Communities. In: *Frontiers in Microbiology*, S. 3445.

Pathan, Shamina Imran; Větrovský, Tomáš; Giagnoni, Laura; Datta, Rahul; Baldrian, Petr; Nannipieri, Paolo; Renella, Giancarlo (2018): Microbial expression profiles in the rhizosphere of two maize lines differing in N use efficiency. In: *Plant and Soil* 433 (1), S. 401–413.

Peiffer, Jason A.; Spor, Aymé; Koren, Omry; Jin, Zhao; Tringe, Susannah Green; Dangl, Jeffery L. et al. (2013): Diversity and heritability of the maize rhizosphere microbiome under field conditions. In: *Proceedings of the National Academy of Sciences* 110 (16), S. 6548–6553.

Pii, Youry; Borruso, Luigimaria; Brusetti, Lorenzo; Crecchio, Carmine; Cesco, Stefano; Mimmo, Tanja (2016): The interaction between iron nutrition, plant species and soil type shapes the rhizosphere microbiome. In: *Plant Physiology and Biochemistry* 99, S. 39–48.

Pivato, Barbara; Semblat, Amélie; Guégan, Thibault; Jacquiod, Samuel; Martin, Juliette; Deau, Florence et al. (2021): Rhizosphere bacterial networks, but not diversity, are impacted by pea-wheat intercropping. In: *Frontiers in Microbiology* 12, S. 1351.

Prasannakumar, M. K.; Netravathi, L. M.; Mahesh, H. B.; Buela Parivallal, P.; Puneeth, M. E.; Sathish, A. et al. (2021): Comparative metagenomic analysis of rice soil samples revealed the diverse microbial population and biocontrol organisms against plant pathogenic fungus Magnaporthe oryzae. In: *3 Biotech* 11 (5), S. 1–11.

Qi, Yueling; Ossowicki, Adam; Yang, Xiaomei; Lwanga, Esperanza Huerta; Dini-Andreote, Francisco; Geissen, Violette; Garbeva, Paolina (2020): Effects of plastic mulch film residues on wheat rhizosphere and soil properties. In: *Journal of Hazardous Materials* 387, S. 121711.

Qiao, Cece; Penton, C. Ryan; Xiong, Wu; Liu, Chao; Wang, Roufei; Liu, Zhengyang et al. (2019): Reshaping the rhizosphere microbiome by bio-organic amendment to enhance crop yield in a maize-cabbage rotation system. In: *Applied soil ecology* 142, S. 136–146.

Raj, Garima; Shadab, Mohammad; Deka, Sujata; Das, Manashi; Baruah, Jilmil; Bharali, Rupjyoti; Talukdar, Narayan C. (2019): Seed interior microbiome of rice genotypes indigenous to three agroecosystems of Indo-Burma biodiversity hotspot. In: *BMC genomics* 20 (1), S. 1–16.

Rascovan, Nicolás; Carbonetto, Belén; Perrig, Diego; Díaz, Marisa; Canciani, Wilter; Abalo, Matías et al. (2016): Integrated analysis of root microbiomes of soybean and wheat from agricultural fields. In: *Scientific reports* 6 (1), S. 1–12.

Reid, Tessa E.; Kavamura, Vanessa N.; Abadie, Maïder; Torres-Ballesteros, Adriana; Pawlett, Mark; Clark, Ian M. et al. (2021): Inorganic chemical fertilizer application to wheat reduces the abundance of putative plant growth-promoting rhizobacteria. In: *Frontiers in Microbiology*, S. 458.

Ridout, Mary E.; Schroeder, Kurtis L.; Hunter, Samuel S.; Styer, James; Newcombe, George (2019): Priority effects of wheat seed endophytes on a rhizosphere symbiosis. In: *Symbiosis* 78 (1), S. 19–31.

Robertson-Albertyn, Senga; Alegria Terrazas, Rodrigo; Balbirnie, Katharin; Blank, Manuel; Janiak, Agnieszka; Szarejko, Iwona et al. (2017): Root hair mutations displace the barley rhizosphere microbiota. In: *Frontiers in plant science* 8, S. 1094.

Roman-Reyna, Veronica; Pinili, Dale; Borja, Frances N.; Quibod, Ian L.; Groen, Simon C.; Alexandrov, Nickolai et al. (2020): Characterization of the leaf microbiome from whole-genome sequencing data of the 3000 rice genomes project. In: *Rice* 13 (1), S. 1–8.

Rossmann, Maike; Perez-Jaramillo, Juan E.; Kavamura, Vanessa N.; Chiaramonte, Josiane B.; Dumack, Kenneth; Fiore-Donno, Anna Maria et al. (2020): Multitrophic interactions in the rhizosphere microbiome of wheat: from bacteria and fungi to protists. In: *FEMS microbiology ecology* 96 (4), fiaa032.

Rothenberg, Sarah E.; Anders, Merle; Ajami, Nadim J.; Petrosino, Joseph F.; Balogh, Erika (2016): Water management impacts rice methylmercury and the soil microbiome. In: *Science of The Total Environment* 572, S. 608–617.

Rüger, Lioba; Feng, Kai; Dumack, Kenneth; Freudenthal, Jule; Chen, Yan; Sun, Ruibo et al. (2021): Assembly patterns of the rhizosphere microbiome along the longitudinal root axis of maize (Zea mays L.). In: *Frontiers in Microbiology* 12, S. 237.

Santos, Susana Silva; Rask, Klara Andrés; Vestergård, Mette; Johansen, Jesper Liengaard; Priemé, Anders; Frøslev, Tobias Guldberg et al. (2021): Specialized microbiomes facilitate natural rhizosphere microbiome interactions counteracting high salinity stress in plants. In: *Environmental and Experimental Botany* 186, S. 104430.

Santos-Medellín, Christian; Edwards, Joseph; Liechty, Zachary; Nguyen, Bao; Sundaresan, Venkatesan (2017): Drought stress results in a compartment-specific restructuring of the rice root-associated microbiomes. In: *MBio* 8 (4), e00764-17.

Sapkota, Rumakanta; Jørgensen, Lise N.; Nicolaisen, Mogens (2017): Spatiotemporal variation and networks in the mycobiome of the wheat canopy. In: *Frontiers in plant science* 8, S. 1357.

Saravanakumar, Kandasamy; Li, Yaqian; Yu, Chuanjin; Wang, Qiang-qiang; Wang, Meng; Sun, Jianan et al. (2017): Effect of Trichoderma harzianum on maize rhizosphere microbiome and biocontrol of Fusarium Stalk rot. In: *Scientific reports* 7 (1), S. 1–13.

Schlatter, Daniel C.; Kahl, Kendall; Carlson, Bryan; Huggins, David R.; Paulitz, Timothy (2020a): Soil acidification modifies soil depth-microbiome relationships in a no-till wheat cropping system. In: *Soil Biology and Biochemistry* 149, S. 107939.

Schlatter, Daniel C.; Yin, Chuntao; Hulbert, Scot; Paulitz, Timothy C. (2020b): Core rhizosphere microbiomes of dryland wheat are influenced by location and land use history. In: *Applied and environmental microbiology* 86 (5), e02135-19.

Schmidt, Jennifer E.; Rodrigues, Jorge L. Mazza; Brisson, Vanessa L.; Kent, Angela; Gaudin, Amélie C. M. (2020): Impacts of directed evolution and soil management legacy on the maize rhizobiome. In: *Soil Biology and Biochemistry* 145, S. 107794.

Semenov, Mikhail V.; Krasnov, George S.; Semenov, Vyacheslav M.; van Bruggen, Ariena H. C. (2020): Long-term fertilization rather than plant species shapes rhizosphere and bulk soil prokaryotic communities in agroecosystems. In: *Applied soil ecology* 154, S. 103641.

Shao, Jiahui; Miao, Youzhi; Liu, Kaiming; Ren, Yi; Xu, Zhihui; Zhang, Nan et al. (2021): Rhizosphere microbiome assembly involves seed-borne bacteria in compensatory phosphate solubilization. In: *Soil Biology and Biochemistry* 159, S. 108273.

Shen, Minchong; Li, Jiangang; Dong, Yuanhua; Zhang, Zhengkun; Zhao, Yu; Li, Qiyun et al. (2021): The Effects of Microbial Inoculants on Bacterial Communities of the Rhizosphere Soil of Maize. In: *Agriculture* 11 (5), S. 389.

Shenton, Matthew; Iwamoto, Chie; Kurata, Nori; Ikeo, Kazuho (2016): Effect of wild and cultivated rice genotypes on rhizosphere bacterial community composition. In: *Rice* 9 (1), S. 1–11.

Shi, Shaohua; Tian, Lei; Nasir, Fahad; Li, Xiujun; Li, Weiqiang; Tran, Lam-Son Phan; Tian, Chunjie (2018a): Impact of domestication on the evolution of rhizomicrobiome of rice in response to the presence of Magnaporthe oryzae. In: *Plant Physiology and Biochemistry* 132, S. 156–165.

Shi, Yu; Li, Yuntao; Xiang, Xingjia; Sun, Ruibo; Yang, Teng; He, Dan et al. (2018b): Spatial scale affects the relative role of stochasticity versus determinism in soil bacterial communities in wheat fields across the North China Plain. In: *Microbiome* 6 (1), S. 1–12.

Silva, Ubiana C.; Medeiros, Julliane D.; Leite, Laura R.; Morais, Daniel K.; Cuadros-Orellana, Sara; Oliveira, Christiane A. et al. (2017): Long-term rock phosphate fertilization impacts the microbial communities of maize rhizosphere. In: *Frontiers in Microbiology* 8, S. 1266.

Singha, K. Malabika; Singh, Brahmanand; Pandey, Piyush (2021): Host specific endophytic microbiome diversity and associated functions in three varieties of scented black rice are dependent on growth stage. In: *Scientific reports* 11 (1), S. 1–17.

Song, Li; Pan, Zhenzhi; Dai, Yi; Chen, Lin; Zhang, Li; Liao, Qilin et al. (2021): High-throughput sequencing clarifies the spatial structures of microbial communities in cadmium-polluted rice soils. In: *Environmental Science and Pollution Research* 28 (34), S. 47086–47098.

Sun, Weimin; Xiao, Enzong; Pu, Zilun; Krumins, Valdis; Dong, Yiran; Li, Baoqin; Hu, Min (2018): Paddy soil microbial communities driven by environment-and microbe-microbe interactions: a case study of elevation-resolved microbial communities in a rice terrace. In: *Science of The Total Environment* 612, S. 884–893.

Sun, Xiang; Kosman, Evsey; Sharon, Amir (2020): Stem endophytic mycobiota in wild and domesticated wheat: structural differences and hidden resources for wheat improvement. In: *Journal of Fungi* 6 (3), S. 180.

Szoboszlay, Márton; Näther, Astrid; Mullins, Ewen; Tebbe, Christoph C. (2019): Annual replication is essential in evaluating the response of the soil microbiome to the genetic modification of maize in different biogeographical regions. In: *PloS one* 14 (12), e0222737.

Taheri, Ahmad Esmaeili; Hamel, Chantal; Gan, Yantai (2015): Pyrosequencing reveals the impact of foliar fungicide application to chickpea on root fungal communities of durum wheat in subsequent year. In: *Fungal ecology* 15, S. 73–81.

Tan, Wenjun; Wang, Junman; Bai, Wenqing; Qi, Jiejun; Chen, Weimin (2020): Soil bacterial diversity correlates with precipitation and soil pH in long-term maize cropping systems. In: *Scientific reports* 10 (1), S. 1–12.

Tao, Jiemeng; Liu, Xueduan; Liang, Yili; Niu, Jiaojiao; Xiao, Yunhua; Gu, Yabing et al. (2017): Maize growth responses to soil microbes and soil properties after fertilization with different green manures. In: *Applied Microbiology and Biotechnology* 101 (3), S. 1289–1299.

Tian, Dagang; Chen, Zaijie; Lin, Yan; Liang, Tingmin; Chen, Ziqiang; Guo, Xinrui et al. (2021a): The Interaction between rice genotype and Magnaporthe oryzae regulates the assembly of rice root-associated microbiota. In: *Rice* 14 (1), S. 1–15.

Tian, Lei; Wang, Enze; Lin, Xiaolong; Ji, Li; Chang, Jingjing; Chen, Hongping et al. (2021b): Wild rice harbors more root endophytic fungi than cultivated rice in the F1 offspring after crossbreeding. In: *BMC genomics* 22 (1), S. 1–12.

Tkacz, Andrzej; Pini, Francesco; Turner, Thomas R.; Bestion, Eloïne; Simmonds, James; Howell, Phil et al. (2020): Agricultural selection of wheat has been shaped by plant-microbe interactions. In: *Frontiers in Microbiology* 11, S. 132.

Tracanna, Vittorio; Ossowicki, Adam; Petrus, Marloes L. C.; Overduin, Sam; Terlouw, Barbara R.; Lund, George et al. (2021): Dissecting disease-suppressive rhizosphere microbiomes by functional amplicon sequencing and 10× metagenomics. In: *MSystems* 6 (3), e01116-20.

Turner, Thomas R.; Ramakrishnan, Karunakaran; Walshaw, John; Heavens, Darren; Alston, Mark; Swarbreck, David et al. (2013): Comparative metatranscriptomics reveals kingdom level changes in the rhizosphere microbiome of plants. In: *The ISME journal* 7 (12), S. 2248–2258.

Ujvári, Gergely; Borsodi, Andrea K.; Megyes, Melinda; Mucsi, Márton; Szili-Kovács, Tibor; Szabó, Attila et al. (2020): Comparison of soil bacterial communities from juvenile maize plants of a long-term monoculture and a natural grassland. In: *Agronomy* 10 (3), S. 341.

Uksa, Marie; Buegger, Franz; Gschwendtner, Silvia; Lueders, Tillmann; Kublik, Susanne; Kautz, Timo et al. (2017): Bacteria utilizing plant‐derived carbon in the rhizosphere of Triticum aestivum change in different depths of an arable soil. In: *Environmental microbiology reports* 9 (6), S. 729–741.

van Deynze, Allen; Zamora, Pablo; Delaux, Pierre-Marc; Heitmann, Cristobal; Jayaraman, Dhileepkumar; Rajasekar, Shanmugam et al. (2018): Nitrogen fixation in a landrace of maize is supported by a mucilage-associated diazotrophic microbiota. In: *PLoS biology* 16 (8), e2006352.

Vescio, Rosa; Malacrinò, Antonino; Bennett, Alison E.; Sorgonà, Agostino (2021): Single and combined abiotic stressors affect maize rhizosphere bacterial microbiota. In: *Rhizosphere* 17, S. 100318.

Visioli, Giovanna; Sanangelantoni, Anna Maria; Vamerali, Teofilo; Dal Cortivo, Cristian; Blandino, Massimo (2018): 16S rDNA profiling to reveal the influence of seed-applied biostimulants on the rhizosphere of young maize plants. In: *Molecules* 23 (6), S. 1461.

Wagner, Maggie R.; Roberts, Joseph H.; Balint‐Kurti, Peter; Holland, James B. (2020): Heterosis of leaf and rhizosphere microbiomes in field‐grown maize. In: *New Phytologist* 228 (3), S. 1055–1069.

Wallace, Jason G.; Kremling, Karl A.; Kovar, Lynsey L.; Buckler, Edward S. (2018): Quantitative genetics of the maize leaf microbiome. In: *Phytobiomes Journal* 2 (4), S. 208–224.

Walsh, Corinne M.; Becker-Uncapher, Isadore; Carlson, Madeline; Fierer, Noah (2021): Variable influences of soil and seed-associated bacterial communities on the assembly of seedling microbiomes. In: *The ISME journal* 15 (9), S. 2748–2762.

Wang, Chaonan; Qin, Yifan; Li, Yilong; Wu, Ruilin; Zhu, Dongqiang; Zhou, Feng; Xu, Fuliu (2021a): Variations of root-associated bacterial cooccurrence relationships in paddy soils under chlorantraniliprole (CAP) stress. In: *Science of The Total Environment* 779, S. 146247.

Wang, Juan; Chapman, Stephen J.; Ye, Qingfu; Yao, Huaiying (2019a): Limited effect of planting transgenic rice on the soil microbiome studied by continuous 13CO2 labeling combined with high-throughput sequencing. In: *Applied Microbiology and Biotechnology* 103 (10), S. 4217–4227.

Wang, Pei; Kong, Xiao; Chen, Hongsong; Xiao, Youlun; Liu, Huijun; Li, Xiaojuan et al. (2021b): Exploration of intrinsic microbial community modulators in the rice endosphere indicates a key role of distinct bacterial taxa across different cultivars. In: *Frontiers in Microbiology*, S. 181.

Wang, Peng; Marsh, Ellen L.; Ainsworth, Elizabeth A.; Leakey, Andrew D. B.; Sheflin, Amy M.; Schachtman, Daniel P. (2017): Shifts in microbial communities in soil, rhizosphere and roots of two major crop systems under elevated CO2 and O3. In: *Scientific reports* 7 (1), S. 1–12.

Wang, Peng; Marsh, Ellen L.; Kruger, Greg; Lorenz, Aaron; Schachtman, Daniel P. (2020): Belowground microbial communities respond to water deficit and are shaped by decades of maize hybrid breeding. In: *Environmental Microbiology* 22 (3), S. 889–904.

Wang, Qingfeng; Jiang, Xin; Guan, Dawei; Wei, Dan; Zhao, Baisuo; Ma, Mingchao et al. (2018a): Long-term fertilization changes bacterial diversity and bacterial communities in the maize rhizosphere of Chinese Mollisols. In: *Applied soil ecology* 125, S. 88–96.

Wang, Runze; Wei, Shuai; Jia, Peihan; Liu, Ting; Hou, Dandi; Xie, Ruohan et al. (2019b): Biochar significantly alters rhizobacterial communities and reduces Cd concentration in rice grains grown on Cd-contaminated soils. In: *Science of The Total Environment* 676, S. 627–638.

Wang, Wenfeng; Zhai, Yanyan; Cao, Lixiang; Tan, Hongming; Zhang, Renduo (2016): Illumina-based analysis of core actinobacteriome in roots, stems, and grains of rice. In: *Microbiological Research* 190, S. 12–18.

Wang, Yu; Zhao, Xu; Guo, Zhiying; Jia, Zhongjun; Wang, Shenqiang; Ding, Kai (2018b): Response of soil microbes to a reduction in phosphorus fertilizer in rice-wheat rotation paddy soils with varying soil P levels. In: *Soil and Tillage Research* 181, S. 127–135.

Wang, Zhishan; Zhu, Yongqiang; Li, Ni; Liu, Hai; Zheng, Huajun; Wang, Weiping; Liu, Yang (2021c): High-throughput sequencing-based analysis of the composition and diversity of endophytic bacterial community in seeds of saline-alkali tolerant rice. In: *Microbiological Research* 250, S. 126794.

Wattenburger, Cassandra J.; Halverson, Larry J.; Hofmockel, Kirsten S. (2019): Agricultural management affects root-associated microbiome recruitment over maize development. In: *Phytobiomes Journal* 3 (4), S. 260–272.

Wen, Xinya; Wang, Meng; Ti, Jingsong; Wu, Yao; Chen, Fu (2017): Bacterial community composition in the rhizosphere of maize cultivars widely grown in different decades. In: *Biology and Fertility of Soils* 53 (2), S. 221–229.

Wen, Zhong-Ling; Yang, Min-Kai; Du, Mei-Hang; Zhong, Zhao-Zhao; Lu, Yun-Ting; Wang, Gu-Hao et al. (2019): Enrichments/derichments of root-associated bacteria related to plant growth and nutrition caused by the growth of an EPSPS-transgenic maize line in the field. In: *Frontiers in Microbiology*, S. 1335.

WEN, Xin-ya; Dubinsky, Eric; Yao, W. U.; Rong, Yu; Fu, CHEN (2016): Wheat, maize and sunflower cropping systems selectively influence bacteria community structure and diversity in their and succeeding crop's rhizosphere. In: *Journal of integrative agriculture* 15 (8), S. 1892–1902.

Wipf, Heidi M. L.; Coleman-Derr, Devin (2021): Evaluating domestication and ploidy effects on the assembly of the wheat bacterial microbiome. In: *PloS one* 16 (3), e0248030.

Wolińska, Agnieszka; Kuźniar, Agnieszka; Gałązka, Anna (2020): Biodiversity in the rhizosphere of selected winter wheat (Triticum aestivum L.) cultivars—genetic and catabolic fingerprinting. In: *Agronomy* 10 (7), S. 953.

Wolna-Maruwka, Agnieszka; Piechota, Tomasz; Niewiadomska, Alicja; Kamiński, Adam; Kayzer, Dariusz; Grzyb, Aleksandra; Pilarska, Agnieszka A. (2021): The effect of biochar-based organic amendments on the structure of soil bacterial community and yield of maize (Zea mays L.). In: *Agronomy* 11 (7), S. 1286.

Wolters, Birgit; Jacquiod, Samuel; Sørensen, Søren J.; Widyasari-Mehta, Arum; Bech, Tina B.; Kreuzig, Robert; Smalla, Kornelia (2018): Bulk soil and maize rhizosphere resistance genes, mobile genetic elements and microbial communities are differently impacted by organic and inorganic fertilization. In: *FEMS microbiology ecology* 94 (4), fiy027.

Wu, Zhaohui; Liu, Qingshu; Li, Zhenyu; Cheng, Wei; Sun, Jimin; Guo, Zhaohui et al. (2018): Environmental factors shaping the diversity of bacterial communities that promote rice production. In: *BMC microbiology* 18 (1), S. 1–11.

Xu, Jiangbing; Zhang, Jianwei; Zhu, Chunwu; Zhu, Jianguo; Lin, Xiangui; Feng, Youzhi (2019): Influence of rice cultivars on soil bacterial microbiome under elevated carbon dioxide. In: *Journal of Soils and Sediments* 19 (5), S. 2485–2495.

Xu, Yan; Ge, Yi; Lou, Yinghua; Meng, Jun; Shi, Lei; Xia, Fang (2021): Assembly strategies of the wheat root-associated microbiome in soils contaminated with phenanthrene and copper. In: *Journal of Hazardous Materials* 412, S. 125340.

Xu, Yan; Ge, Yi; Song, Jianxiao; Rensing, Christopher (2020): Assembly of root-associated microbial community of typical rice cultivars in different soil types. In: *Biology and Fertility of Soils* 56 (2), S. 249–260.

Xuan, Do Thi; Guong, Vo Thi; Rosling, Anna; Alström, Sadhna; Chai, Benli; Högberg, Nils (2012): Different crop rotation systems as drivers of change in soil bacterial community structure and yield of rice, Oryza sativa. In: *Biology and Fertility of Soils* 48 (2), S. 217–225.

Yang, Fenghuan; Zhang, Jie; Zhang, Huaying; Ji, Guanghai; Zeng, Liexian; Li, Yan et al. (2020a): Bacterial blight induced shifts in endophytic microbiome of rice leaves and the enrichment of specific bacterial strains with pathogen antagonism. In: *Frontiers in plant science* 11, S. 963.

Yang, Luhua; Danzberger, Jasmin; Schöler, Anne; Schröder, Peter; Schloter, Michael; Radl, Viviane (2017): Dominant groups of potentially active bacteria shared by barley seeds become less abundant in root associated microbiome. In: *Frontiers in plant science* 8, S. 1005.

Yang, Luhua; Schröder, Peter; Vestergaard, Gisle; Schloter, Michael; Radl, Viviane (2020b): Response of barley plants to drought might be associated with the recruiting of soil-borne endophytes. In: *Microorganisms* 8 (9), S. 1414.

Yao, Youhua; Yao, Xiaohua; An, Likun; Bai, Yixiong; Xie, Deqing; Wu, Kunlun (2020): Rhizosphere bacterial community response to continuous cropping of Tibetan barley. In: *Frontiers in Microbiology*, S. 3017.

Yergeau, Étienne; Quiza, Liliana; Tremblay, Julien (2020): Microbial indicators are better predictors of wheat yield and quality than N fertilization. In: *FEMS microbiology ecology* 96 (2), fiz205.

Yin, Chuntao; Casa Vargas, Juan M.; Schlatter, Daniel C.; Hagerty, Christina H.; Hulbert, Scot H.; Paulitz, Timothy C. (2021a): Rhizosphere community selection reveals bacteria associated with reduced root disease. In: *Microbiome* 9 (1), S. 1–18.

Yin, Chuntao; Mueth, Nicholas; Hulbert, Scot; Schlatter, Daniel; Paulitz, Timothy C.; Schroeder, Kurtis et al. (2017): Bacterial communities on wheat grown under long-term conventional tillage and no-till in the Pacific Northwest of the United States. In: *Phytobiomes Journal* 1 (2), S. 83–90.

Yin, Chuntao; Schlatter, Daniel C.; Kroese, Duncan R.; Paulitz, Timothy C.; Hagerty, Christina H. (2021b): Impacts of lime application on soil bacterial microbiome in dryland wheat soil in the Pacific Northwest. In: *Applied soil ecology* 168, S. 104113.

Yu, Yongjie; Zhang, Jianwei; Petropoulos, Evangelos; Baluja, Marcos Q.; Zhu, Chunwu; Zhu, Jianguo et al. (2018): Divergent responses of the diazotrophic microbiome to elevated CO2 in two rice cultivars. In: *Frontiers in Microbiology* 9, S. 1139.

Zhang, Hao Qing; Zhao, Xue Qiang; Shi, Yu; Liang, Yuting; Shen, Ren Fang (2021a): Changes in soil bacterial communities with increasing distance from maize roots affected by ammonium and nitrate additions. In: *Geoderma* 398, S. 115102.

Zhang, Jingying; Zhang, Na; Liu, Yong-Xin; Zhang, Xiaoning; Hu, Bin; Qin, Yuan et al. (2018): Root microbiota shift in rice correlates with resident time in the field and developmental stage. In: *Science China Life Sciences* 61 (6), S. 613–621.

Zhang, Lu; Ge, An‐Hui; Tóth, Tibor; An, Fenghua; Guo, Liangliang; Nie, Zhaoyang et al. (2021b): Soil bacterial microbiota predetermines rice yield in reclaiming saline‐sodic soils leached with brackish ice. In: *Journal of the Science of Food and Agriculture* 101 (15), S. 6472–6483.

Zhang, Wei-Guo; Wen, Tao; Liu, Li-Zhu; Li, Jiang-Ye; Gao, Yan; Zhu, Dong et al. (2021c): Agricultural land-use change and rotation system exert considerable influences on the soil antibiotic resistome in Lake Tai Basin. In: *Science of The Total Environment* 771, S. 144848.

Zhang, Xiaoxia; Zhang, Ruijie; Gao, Jusheng; Wang, Xiucheng; Fan, Fenliang; Ma, Xiaotong et al. (2017): Thirty-one years of rice-rice-green manure rotations shape the rhizosphere microbial community and enrich beneficial bacteria. In: *Soil Biology and Biochemistry* 104, S. 208–217.

Zhang, Yang; Jiang, Wenzhu; Li, Qing; Xu, Wenjie; Wang, Juanjuan; Hu, Jian; Zhang, Zujian (2021d): Soil nutrient levels determine the variation of bacterial communities in the rhizosphere of rice under different conditions of climate and genotype. In: *Applied soil ecology* 167, S. 104025.

Zhao, Jun; Ni, Tian; Li, Jing; Lu, Qiang; Fang, Zhiying; Huang, Qiwei et al. (2016): Effects of organic–inorganic compound fertilizer with reduced chemical fertilizer application on crop yields, soil biological activity and bacterial community structure in a rice–wheat cropping system. In: *Applied soil ecology* 99, S. 1–12.

Zhou, Xin; Wang, Jin-Ting; Zhang, Zhi-Feng; Li, Wei; Chen, Wen; Cai, Lei (2020a): Microbiota in the rhizosphere and seed of rice from China, with reference to their transmission and biogeography. In: *Frontiers in Microbiology*, S. 995.

Zhou, Yi; Coventry, David R.; Gupta, Vadakattu VSR; Fuentes, David; Merchant, Andrew; Kaiser, Brent N. et al. (2020b): The preceding root system drives the composition and function of the rhizosphere microbiome. In: *Genome biology* 21 (1), S. 1–19.

Zhu, Chen; Ling, Ning; Guo, Junjie; Wang, Min; Guo, Shiwei; Shen, Qirong (2016a): Impacts of fertilization regimes on arbuscular mycorrhizal fungal (AMF) community composition were correlated with organic matter composition in maize rhizosphere soil. In: *Frontiers in Microbiology* 7, S. 1840.

Zhu, Shusheng; Vivanco, Jorge M.; Manter, Daniel K. (2016b): Nitrogen fertilizer rate affects root exudation, the rhizosphere microbiome and nitrogen-use-efficiency of maize. In: *Applied soil ecology* 107, S. 324–333.

Zhu, Yong-Guan; Su, Jian-Qiang; Cao, Zhihong; Xue, Kai; Quensen, John; Guo, Guang-Xia et al. (2016c): A buried Neolithic paddy soil reveals loss of microbial functional diversity after modern rice cultivation. In: *Science bulletin* 61 (13), S. 1052–1060.

Žiarovská, Jana; Medo, Juraj; Kyseľ, Matúš; Zamiešková, Lucia; Kačániová, Miroslava (2020): Endophytic bacterial microbiome diversity in early developmental stage plant tissues of wheat varieties. In: *Plants* 9 (2), S. 266.
